# Unraveling dyadic psycho-physiology of social presence between strangers during an audio drama – a signal-analysis approach

**DOI:** 10.3389/fpsyg.2023.1153968

**Published:** 2023-10-19

**Authors:** Janne Kauttonen, Sander Paekivi, Jaakko Kauramäki, Pia Tikka

**Affiliations:** ^1^Competences, RDI and Digitalization, Haaga-Helia University of Applied Sciences, Helsinki, Finland; ^2^School of Arts, Design and Architecture, Aalto University, Espoo, Finland; ^3^Aalto NeuroImaging, Aalto University, Espoo, Finland; ^4^Max Planck Institute for the Physics of Complex Systems, Dresden, Germany; ^5^Department of Psychology and Logopedics, Faculty of Medicine, University of Helsinki, Helsinki, Finland; ^6^Cognitive Brain Research Unit, Faculty of Medicine, University of Helsinki, Helsinki, Finland; ^7^Enactive Virtuality Lab, Baltic Film, Media and Arts School (BFM), Centre of Excellence in Media Innovation and Digital Culture (MEDIT), Tallinn University, Tallinn, Estonia

**Keywords:** audio narratives, non-verbal dyadic interaction, multimodal data, signal analysis, machine learning, time series analysis, recurrence quantification analysis

## Abstract

A mere co-presence of an unfamiliar person may modulate an individual’s attentive engagement with specific events or situations to a significant degree. To understand better how such social presence affects experiences, we recorded a set of parallel multimodal facial and psychophysiological data with subjects (*N* = 36) who listened to dramatic audio scenes alone or when facing an unfamiliar person. Both a selection of 6 s affective sound clips (IADS-2) followed by a 27 min soundtrack extracted from a Finnish episode film depicted familiar and often intense social situations familiar from the everyday world. Considering the systemic complexity of both the chosen naturalistic stimuli and expected variations in the experimental social situation, we applied a novel combination of signal analysis methods using inter-subject correlation (ISC) analysis, Representational Similarity Analysis (RSA) and Recurrence Quantification Analysis (RQA) followed by gradient boosting classification. We report our findings concerning three facial signals, gaze, eyebrow and smile that can be linked to socially motivated facial movements. We found that ISC values of pairs, whether calculated on true pairs or any two individuals who had a partner, were lower than the group with single individuals. Thus, audio stimuli induced more unique responses in those subjects who were listening to it in the presence of another person, while individual listeners tended to yield a more uniform response as it was driven by dramatized audio stimulus alone. Furthermore, our classifiers models trained using recurrence properties of gaze, eyebrows and smile signals demonstrated distinctive differences in the recurrence dynamics of signals from paired subjects and revealed the impact of individual differences on the latter. We showed that the presence of an unfamiliar co-listener that modifies social dynamics of dyadic listening tasks can be detected reliably from visible facial modalities. By applying our analysis framework to a broader range of psycho-physiological data, together with annotations of the content, and subjective reports of participants, we expected more detailed dyadic dependencies to be revealed. Our work contributes towards modeling and predicting human social behaviors to specific types of audio-visually mediated, virtual, and live social situations.

## 1. Introduction

Dramatized narratives, such as movies, audio stories, and literate texts, have a strong tendency to create intersubjectively shared experiences between people who engage with them. Such experiential situations intertwine the human brain and body with its environment in a holistic manner that can be argued to be systemic and complex by nature (Varela et al., 1991; see Tikka et al., 2023, for review). In this study, we applied a novel data analysis approach that takes into account the systemic complexity of the experimental setting, in line with Turkheimer et al. (2022). The context-dependent time-locked synchronization has been demonstrated for neural signals in various neuroimaging studies using functional magnetic resonance imaging (fMRI) and magnetoencephalography (MEG) when subjects have watched feature films (Hasson et al., 2004; Jääskeläinen et al., 2008; Kauttonen et al., 2015, 2018; Lankinen et al., 2018; Tikka et al., 2018; see Jääskeläinen et al., 2021 for review), recorded dance performance (Jola et al., 2013), or listened to audio narratives (Boldt et al., 2013, 2014; Koskinen and Seppä, 2014; Simony et al., 2016; Koskinen et al., 2020). Synchronization of brain functions across different viewers is more extensive when the stimulus represents socio-emotionally meaningful contexts (e.g., Stephens et al., 2010; Saarimäki, 2021). Yet, in these studies, due to the experimental conditions in neuroimaging labs, the so-called synchronization is to some extent hypothetical, taken that the subjects experience the content alone, and not in real-time interaction with others (e.g., Ames et al., 2014). It can be assumed that the social presence of another person, perhaps even a stranger, affects this synchronization in some way.

To emphasize the presence of another listener, who is unfamiliar to the other participant, we designed an experimental condition where two persons (later, “partners”) were listening to the same audio drama while purposefully unnaturally facing one another. In the control condition a person was listening to the audio drama alone. This setting allowed us to collect facial expressions and a set of psycho-physiological signals from a dramatically contextualized dyadic co-presence between two strangers and compare that data with single listeners (see Methods section for details). During a long-duration behavioral experiment, the attention of a person who is alone conducting the task may shift from task-related thoughts to non-task-related thoughts, a cognitive phenomenon referred to as “mind-wandering” (see Smallwood and Schooler, 2015). In social settings such as in the dyadic listening task where one is facing a stranger, such a shift of attention from the tasks to the other person can be assumed to be more frequent than when listening to a story alone, not only due to the mind-wandering phenomena but also due to the awareness of the attention of the other in the shared situation that may “modulate brain regions involved in regulating behavioral motivation” Chib et al. (2018), thus adding socially induces variations to the complex behavioral dynamics, for instance, enhancing the performance (‘social facilitation’, Zajonc, 1965).

Any socially conditioned situation is characterized by modifications in physiological and motor responses, such as eye-blinks and eye-contact, indicating the shift of attention from the shared object of attention (narrative) to the other person (Nakano, 2015; Chauhan et al., 2017; Shapiro et al., 2017). The co-presence of another may also “influence both the top-down and bottom-up attention-related processes guiding the decision to move the eyes” (Tricoche et al., 2020; for a review, see Stephenson et al., 2021). Shifting one’s gaze to the eyes of the other person might indicate a social action, for instance, a confirmation of joint attention, or a search for a confirmation of a joint affective response (Hamilton, 2016). If the other person is a stranger, eye-contact may also have an important role in establishing a non-verbal agreement of mutual co-presence in the shared space (Miura and Noguchi, 2022). The human skill to recognize and make judgments of an unfamiliar person relies to a great extent on unconscious neural processing of dynamic features of the new face (George, 2016). The presence of another person can be expected to change the properties of the physiological signals of an individual in a complex manner (Chib et al., 2018; Miura and Noguchi, 2022; see Hamilton, 2016; Stephenson et al., 2021, for reviews).

In a face-to-face situation even without verbal interaction, following (Hasson and Frith, 2016), the behavior of the subjects is understood as similarly dynamically coupled, as during a dialogue where interacting parties take turns. The term ‘synchronization’ is applied here in line with Pikovsky et al. (2001) for describing the tendency of connected systems to organize their cognitive oscillations and motor movement together. Importantly, instead of time-locked synchrony of the *same* simultaneous behavior (e.g., imitation), we take the synchrony of signals to describe the presence of some functional relation (coupling) between two *different* states of the coupling systems (Pikovsky et al., 2001). Furthermore, synchrony or coupling may rely on linear, nonlinear, instantaneous, or delayed coupling of two systems.

Dynamical coupling of social signals is considered an elementary part of human-human interaction, such as with synchronized smiles that already babies show (Ruvolo et al., 2015). While computing synchronization mathematically is often straightforward, e.g., using correlation, interpretation of its meaning for physiological signals is far from straightforward. To what extent social synchronization can be assumed to happen between two people, is dependent on the contextual factors and individual personality traits of the co-present persons (Lumsden et al., 2012; Neufeld et al., 2016), detectable in the physiological signals (Stephens et al., 2010; Canento et al., 2011; Soleymani et al., 2012; Kragel and Labar, 2013).

How and when do social interactions tend towards synchrony? While individuals are listening to the same narrative, their personal attitudes or worldviews are to produce some context-related differences. For example, our audio narrative included humorous, tragic, and sexually colored moments. In this case, two persons could interact either through spontaneous alignment in response to the stimulus content (e.g., simultaneously shared humor or embarrassment), in a cognitive synergy in terms of the conducted task of listening (not so much responding to the narrative content but to the joint task), or the situation could elicit a spontaneous coupled response to the other person’s reaction and not so much to the narrative (“I smile because she smiles”). These dramatically “lifted” moments in the narrative could produce observable, significant physiological markers in the data (Stuldreher et al., 2020).

To have more insights to such social systems, we studied the effect of dyadic interactions on the synchronization of individuals’ time-locked response signals, which can take rather complex forms. Addressing dynamical modifications of contextually situated human interaction from holistic complex systems point of view called for application of a range of computational methodologies, incorporating signal analysis and machine learning (ML) techniques. We investigated how the co-presence of an unfamiliar individual modulates attentive and physiological responses during shared audio narrative experiences. To facilitate this, we employed conventional tools based on linear relationships, such as correlation and normality assuming tests, as well as non-linear tools such as recurrence quantification and classification algorithms. By employing a multi-methodological approach, we offer new insights into the complexities of social synchronization and individual variability in shared contexts.

To initiate the investigation of the dynamical differences between two groups of data and to further demonstrate the suitability of methods employed, we posed two hypotheses. Firstly, we hypothesized that the paired setup would modulate the physiological signals in a way that would be detected in the higher synchrony (e.g., correlation) of signals between paired subjects (e.g., simultaneously smiling) compared to subjects, who listen to the narrative alone. Based on data from the body of neuroscientific literature, most likely, the single listener group could be expected to result with higher intersubject synchrony than the paired group as the latter group would present more behavioral variations due to the unavoidable social interaction in the experimental setting. However, ISC may not allow such high temporal resolution that would allow detecting the possible delayed responses of the individuals of the pairs to the facial (“dialogical”) behavior of the partner, thus any temporally close shared facial behaviors (gaze, smile, eye-brow movements) of the pairs could theoretically show higher synchrony. Due to these possibilities, although unlikely, we chose to test the hypothesis that the paired setup would show higher intersubject synchrony than the single listener setup; a negative correlation coefficient would support the opposite version. Secondly, we hypothesized that signals obtained from our paired setup would exhibit more distinctive and dynamically rich variations compared to those from a single-subject setup. In the latter, we expected the signals to be primarily driven by auditory stimuli, rather than the interactive dynamics present in the paired setting. Here richer dynamics of pairs correspond to increased specific types of activity and variability in the signal. Based on these assumptions, we further made a third hypothesis that one can identify signals recorded from paired and individual subjects based on their signal segments recurrent properties.

### 1.1. Meeting the methodic challenges of signal analysis of dyadic coupling

The interest in identification of physiological signals related to distinct mental phenomena, such as emotions, has generated a range of methodical approaches (e.g., Stephens et al., 2010; Canento et al., 2011; Soleymani et al., 2012; Kragel and Labar, 2013). For addressing the complex nature of the cognitive phenomena under study, we employed ISC and RSA methods with two novel additions. First, a sliding-window based scan type RSA analysis of the audio drama, and secondly a combination of RQA and machine learning, to provide a deeper understanding of the effect of dyadic interaction on the subjects.

The inter-subject correlation analysis (ISC; Hasson et al., 2004; Nastase et al., 2019) has become a standard method particularly in the field of neuroscience as it can be applied even when the generating process of the signal is unknown. ISC assesses the similarity (often referred to as synchrony) of time series from separate subjects exposed to the same time-locked paradigm, typically using Pearson correlation metrics, which is a linear measure of synchrony. ISC can be further leveraged in Representational Similarity Analysis (RSA; Kriegeskorte et al., 2008), a method for comparing groups of pairwise similarity measures. The main limitation of ISC is, however, that it requires two time-locked signals from different individuals and cannot be applied to an individual signal.

To analyze individual signals, we applied Recurrence Quantification Analysis (RQA), formalized by Zbilut and Webber (1992), and expanded on over the years (see, e.g., Marwan et al., 2007, for overview). Recurrence is an important feature of dynamic systems, characterizing repetitions of values or ranges of values, whose frequencies reveal behavioral regimes of stochastic signals, without any assumption of an underlying model. Quantifying recurrent patterns in signals has been found useful in extracting distinguishing and descriptive information from dynamic systems (Webber and Marwan, 2015), including physiological signals (Fusaroli et al., 2014; Guevara et al., 2017; Xu et al., 2020; Stevanovic et al., 2021). This information can be further applied in building features for predictive machine learning analysis.

We demonstrate the effectiveness of RQA as a fundamental research tool into the effect dyadic interaction has on complex recurrent patterns in facial response signals. With predictive power being the benchmark, the impact and order of importance of RQA quantifiers reveals prioritization of complex behaviors for this target variable, which in turn can tell us much about the interactions taking place in dyadic interactions. This approach is (to the author’s knowledge) novel in the field and enabled specifically by the interpretability of recurrence quantifiers, and as such makes up a core motivation of this study. Our analyses do not rely on manual annotations, hence they can be applied in data-driven exploration of dyadic physiological data.

## 2. Materials and methods

### 2.1. Subjects

All subjects signed an informed consent prior to participation. The study was approved by the local institute ethics committee (Aalto University). Subjects (*N* = 36; 20–47 years, mean 27.5) were all female, and spoke Finnish as their native language. The subjects were divided into two equal-sized groups by the experimenters depending on subject availability, and they either participated in the experiment alone (“subject single,” denoted as SS) or together with another participant (“subject paired,” denoted as SP) where we employed a hyperscanning paradigm, collecting data simultaneously from both participants. The experiment type was not informed to the subjects prior to their arrival to the experiment site.

### 2.2. Measurement setup

**Figure 1:**
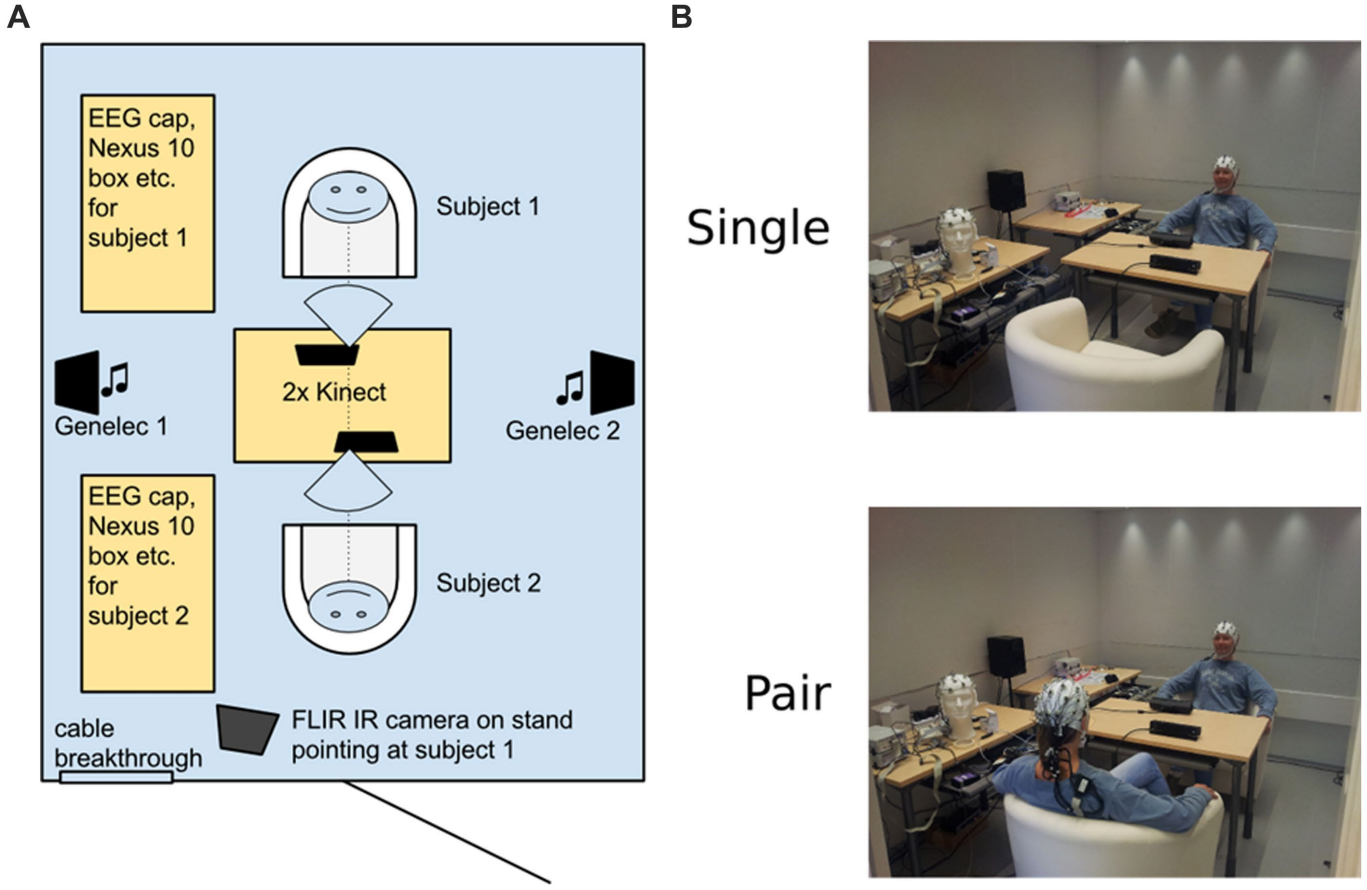
The measurement room setup. **(A)** Schematic diagram of the room and placement of periphery. **(B)** Exemplary photos of the actual measurement room in the two different conditions (one of the authors working as a model). The set-up image shows one of the authors.

After informing of the procedures, the subjects were guided to the measurement room. The room, depicted in Figure 1, was 2.5 m by 3.2 m in size, soundproof, well-lit, and included a central table with two chairs on opposite sides. The two high-quality loudspeakers (Genelec 2029A monitors, Genelec Oy, Iisalmi, Finland) were located symmetrically beside the side walls with equal distance to both subjects (~1.2 m from subjects’ ears). The central table housed the two Kinect 2.0 sensors (Microsoft, WA, USA), directed to both subjects, but allowing direct eye contact in the paired condition without interference. In the single subject setup, the subject was seated in “Subject 1″ position, opposite to the door. Physiological measures and depth + traditional video streams were collected via Kinect. In addition, our setup allowed collection of EEG, respiration, galvanic skin response and infrared video data. In noting, due to the limitations of the space, and for not complicating the explication of the method, we decided to exclude this data from the current paper. The collected multisource data is reserved for further validation of the proposed method in the future.

The subjects were instructed in the audiotrack to sit comfortably but relatively still, attend to the auditory stimuli, and at the same time, either pay attention (1) their feelings during the “single” condition, (2) to the other subject’s feelings in the “paired” condition without talking. Instructions were pre-recorded to minimize experimental variability, and to allow the subjects to familiarize with the setup, and to allow the experimenters to verify smooth collection of input signals in the control room before the actual experiment sounds. These instructions were used to encourage the subjects to have eye contact with the other participant and not acting such that it would disturb data collection (e.g., talk aloud, do major change in position or stance). During single condition, there was a fixation mark on the opposite wall, roughly at the level of the other subject’s head during paired condition, and the subjects were instructed to keep their head fixed to that direction.

### 2.3. Stimuli

The whole setup consisted of a pre-recorded set of trigger sounds, instructions, affective sound localizer (10 clips) and an excerpt of 27-min soundtrack from a Finnish episode film “Kohtaamisia” (dir. Saara Cantell). The audio parts were presented at 16-bit, 44,100 Hz using a dedicated computer with Presentation program (v17.2, Neurobehavioral systems, Albany, CA, USA), sending digital triggers to Nexus-10 devices. Besides digital triggers, we used short trigger sounds both at the start and in the end to allow manual video synchronization. The sound level was fixed to a comfortable listening level, peaking at about 75 dB SPL at the loudest portions, and was kept constant across subjects. After a one-minute instruction period, the experiment continued automatically to the localizer part. The waveform of the whole stimulus is depicted in Supplementary Figure S1 using both linear and decibel scale.

We used a subset of 10 sounds from the IADS-2 affective sound dataset (Bradley and Lang, 2007) as a reference stimulus, which were played before the audio drama. We selected the 10 sounds to have examples of extreme valence and arousal values of the whole dataset continuum. The IADS-2 sound codes were 110, 424, 817, 286, 216, 261, 246, 262, 810, and 277, presented in this order. Content of these sounds were: [Child’s laughter], [Car crash], [African music], [Man yelling before sounds of shooting], [Making love], [Child crying], [Heartbeat], [Yawning], [Classical instrumental music], and [Woman screaming]. These sounds, originally edited to 6 s, were each looped to a total of 12 s presentation time to allow time for the physiological signal changes. After each presentation of IADS-2 sound, there was a 10 s silent period. The segments were characterized by different socio-emotional content and serve in this paper as an informative introduction to the ISC analysis.

After all the IADS-2 sounds, the experiment continued directly to the soundtrack phase. Notably, the soundtrack was presented in stereo, and on certain occasions the sounds came dominantly from either speaker, possibly promoting joint attention of the subjects.

### 2.4. Data collection

#### 2.4.1. Video data

We collected both 512 × 424 pixel resolution depth video and 960 × 540 color video at 30 Hz from the one or two Kinect sensors. Further, we included microphone signals to allow exact synchronization of the video onset. The data were initially stored using iPI recorder software (iPi Soft LLC, Russia) in a proprietary video format, and later exported to standard MP4 video. Again, we employed two independent computers for the recording due to bandwidth requirements of the Kinect video stream.

#### 2.4.2. Web ratings

We asked the subjects to rate the soundtrack, at their own pace, using an online web-based tool. The rating was done by intuitive up/down mouse cursor movements while listening to the audio drama. Subjects annotated both valence and arousal-related experiences. The web rating tool records the movements at a 5 Hz sampling rate, allowing us to get a continuous rating of the long soundtrack.

#### 2.4.3. Expert annotations

Audio drama content was annotated by an external expert using ELAN annotation tool (Max Planck Institute^1^).

### 2.5. Signal processing and feature extraction

#### 2.5.1. Video (RGB, IR)

After the Kinect video collection, we first exported the whole recording soundtrack as plain audio. The soundtrack was used to extract the onset and offset of the video recording based on audio trigger sounds, and this information (video frame numbers) was used to export color/RGB video from the proprietary combined depth + color video for further processing.

#### 2.5.2. Facial muscle activity

**Figure 2:**
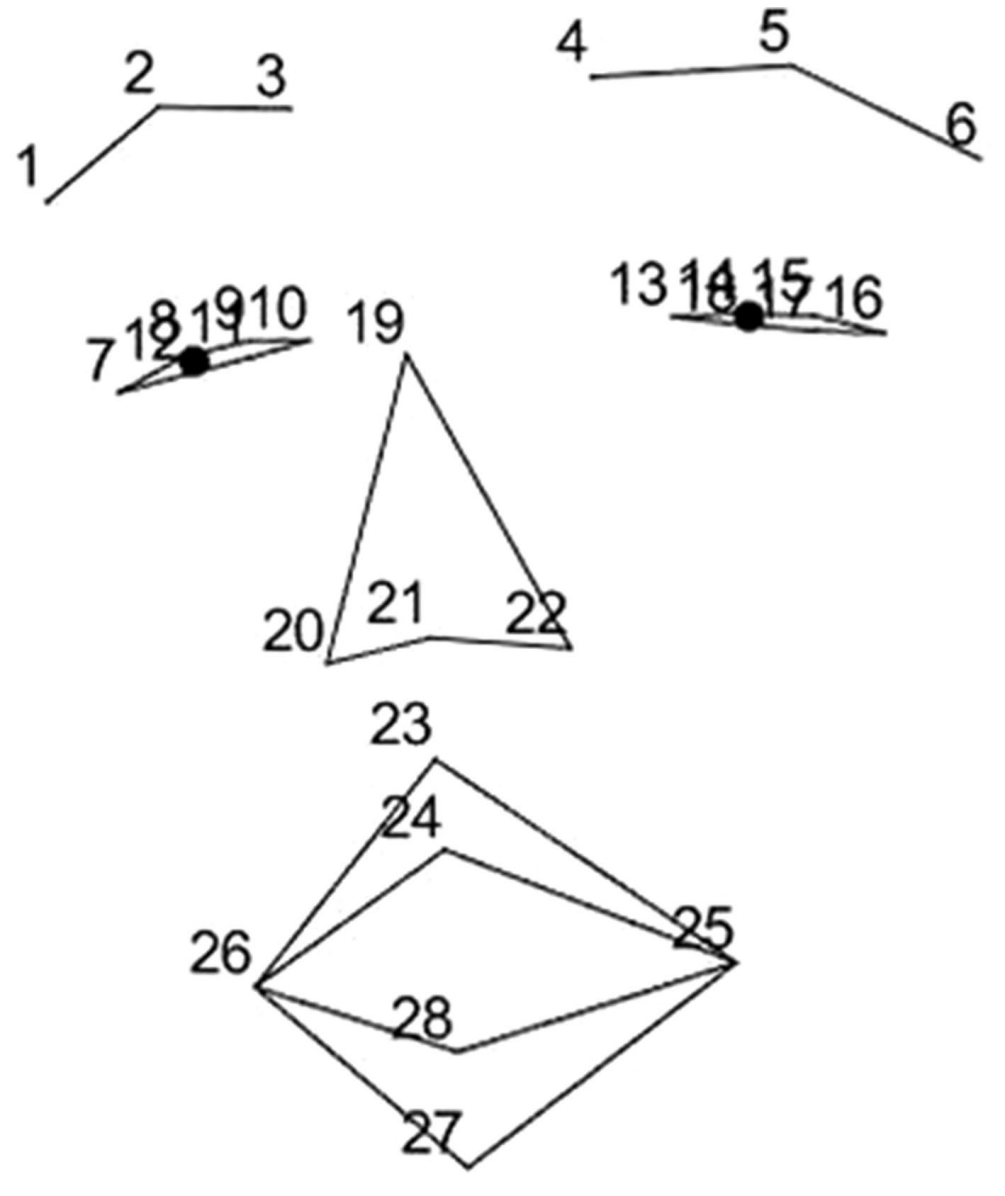
Facial landmarks as they were captured from videos.

Facial activity is typically measured in terms of Facial Action Coding System (FACS) (Ekman and Friesen, 1978). Originally, FACS was developed as a tool for manually quantifying observed facial actions, but it has turned out to be useful in automatic facial expression detection as well. We used the IntraFace software (De la Torre et al., 2015) for detecting the movement of a set of landmarks on the face and performed an approximate FACS coding based on distances between the landmarks. Figure 2 depicts the numbers and locations of FACS, which are listed in Table 1 with the corresponding muscles involved.

**Table 1:** Action units in facial analysis.

**AU**	**Name**	**Muscle**	**Landmarks**
AU 1	Inner Brow Raiser	Frontalis, pars medialis	3, 10, 4, 13
AU 2	Outer Brow Raiser	Frontalis, pars lateralis	1, 7, 6, 16
AU 4	Brow Lowerer	Corrugator supercilii	3, 4
AU 5	Upper Lid Raiser	Levator palpebrae superioris	8, 9, 11, 12, 14, 15, 17, 18
AU 6	Cheek Raiser	Orbicularis oculi	8, 9, 11, 12, 14, 15, 17, 18
AU 9	Nose Wrinkler	Levator labii	10, 13, 20, 22
AU 12	Lip Corner Puller	Zygomaticus major	25, 26
AU 18	Lip Puckerer	Incisivii labii	25, 26
AU 25	Lips part	(several muscles)	24, 28

Landmarks are visualized in Figure 2.

Along with the facial feature extraction, IntraFace software provided gaze angle estimation from the video. The video was obtained with a Kinect wide angle lens, so the accuracy was not comparable to for instance an eye-tracking device that processes a more defined video stream, often taken with a dedicated telephoto lens. Due to this, for instance, direct eye contact with the other participant in a paired setup could not be estimated with certainty. Still, as the angle was a continuous measure of interest that seemed robust enough, we chose it for further analysis.

### 2.6. Signal analysis

Our analysis methods of choice were Inter-Subject Correlation (ISC) analysis, Representational Similarity Analysis (RSA) and gradient boosting classification (CatBoost algorithm) combined with Recurrence Quantification Analysis (RQA) as methods to see how individual listeners and those who listened with pairs differed. First, we focus on describing ISC and RSA (2.6.1) aimed to check the hypothesis of differences between the single and paired groups, using a sliding-window based scan type RSA analysis of the audio drama. The second section focuses on RSA and ML (2.6.2) aimed to check the hypothesis of differences between the single and paired groups, using a combination of RQA and ML to provide a deeper understanding of the effect of dyadic interaction on the subjects.

#### 2.6.1. Representational similarity analysis and inter-subject correlations

RSA is an approach to visualize and quantify complex time-series data, irrespective of underlying models, based on a measure of distance describing the similarity between two time-series. We used the ISC as this distance measure, which is based on interpreting the signal as made up of three components: c(t) reflecting processing triggered by the stimulus (the audio track), and should be consistent across subjects; $id_{A}\left(t\right)$ reflects the idiosyncratic response of subject *A* (respectively for the other subject, *B*); $\varepsilon_{A}\left(t\right)$ is an error term, reflecting spontaneous activity unrelated to either the stimulus or subject-specific response (Nastase et al., 2019). For a time-series segment, the signal $x_{A}\left(t\right)$ is given then as:


$$x_{A}\left(t\right)=\alpha \;c\left(t\right)+\beta \;id_{A}\left(t\right)+\varepsilon_{A}\left(t\right)$$

ISC then assumes that as the second subject *B* experiences the same, time-locked stimulus (e.g., a movie), their same-source signals will also be a mixture of c , $id_{B}$ and $\varepsilon_{B}$ . The component c(t) then should be perfectly correlated for subjects *A* and *B*, while id(t) and ε(t) will not (Nastase et al., 2019). The Pearson correlation $r\left(x_{A}x_{B}\right)$ between the signals of subjects *A* and *B* then will increase with α and the average r is a proxy for the latter. Thus, ISC filters out subject-specific information and reveals the joint stimulus-induced component.

Between all individuals, paired or unpaired, we calculated a similarity measure based on the Pearson correlation coefficient, given simply as $d_{P}=1+r$ . While this is just a shift of the regular correlation coefficient, it has been used as such in RSA (Kriegeskorte et al., 2008; Nili et al., 2014) previously and we keep the same convention for consistency. The measure as such is called the correlation distance, where the values can be read as $d_{P}=0$ indicates strong dissimilarity, $d_{P}=2$ indicates strong similarity, with $d_{P}=1$ meaning r=0 and thus indicating no negative or positive correlation. This way, an ISC matrix gives a quantifiable and visually interpretable result to determine differences between individual subjects and differences between groups of subjects. We are particularly interested in the difference of ISCs between the group of individual subjects and the group of paired subjects.

In RSA analysis, pair-wise ISC values resemble a time point Representational Dissimilarity Matrix (RDM), where each cell contains the ISC reflecting the respective similarity or dissimilarity between the individuals. From the RDMs, one can gather a visual overview of similarity patterns associated with the individuals, by conditions (specific emotional content, annotation) and modalities (different modes of response, i.e., time-series) (Kriegeskorte et al., 2008; Nili et al., 2014). A further comparison can then be carried out on separate RDMs via for example the Kendall’s Tau correlation coefficient, enabling one to identify narrative inputs that produce similar RDMs within or outside of measurement modalities. Kendall’s Tau correlation is a non-parametric measure of similarity and it’s a recommended method to be applied with RDMs (Nili et al., 2014). By comparing RDMs of different modalities, we could identify stimuli that produce similar, in structure and magnitude, responses in the subjects.

In this work, we performed RSA using specific windows of signals for localizers and applying a sliding window method for audio drama. In the latter, the full-length signals are first analyzed by computing RSA matrices in short (20s) overlapping windows, and then tested against a model matrix with the respective statistical significance filtered by the false discovery rate (Benjamini and Hochberg, 1995) procedure. The former provides a baseline understanding of the intuitive meaning of ISC and RSA analysis, while the latter reveals a general understanding of the relations between audio drama narrative content and ISCs.

Based on data from the body of literature (section #), one may assume that the paired and the single listener groups would show differences in synchrony when compared. However, for arbitrary testing using RSA, we hypothesized detecting an increased correlation of signals in the paired setup when compared against single listeners. That is, in the models that the RDM is compared against, ISC values for the SP group are assumed higher than those for SS or mixed SS-SP subject groups.

#### 2.6.2. Recurrence quantification analysis

**Figure 3:**
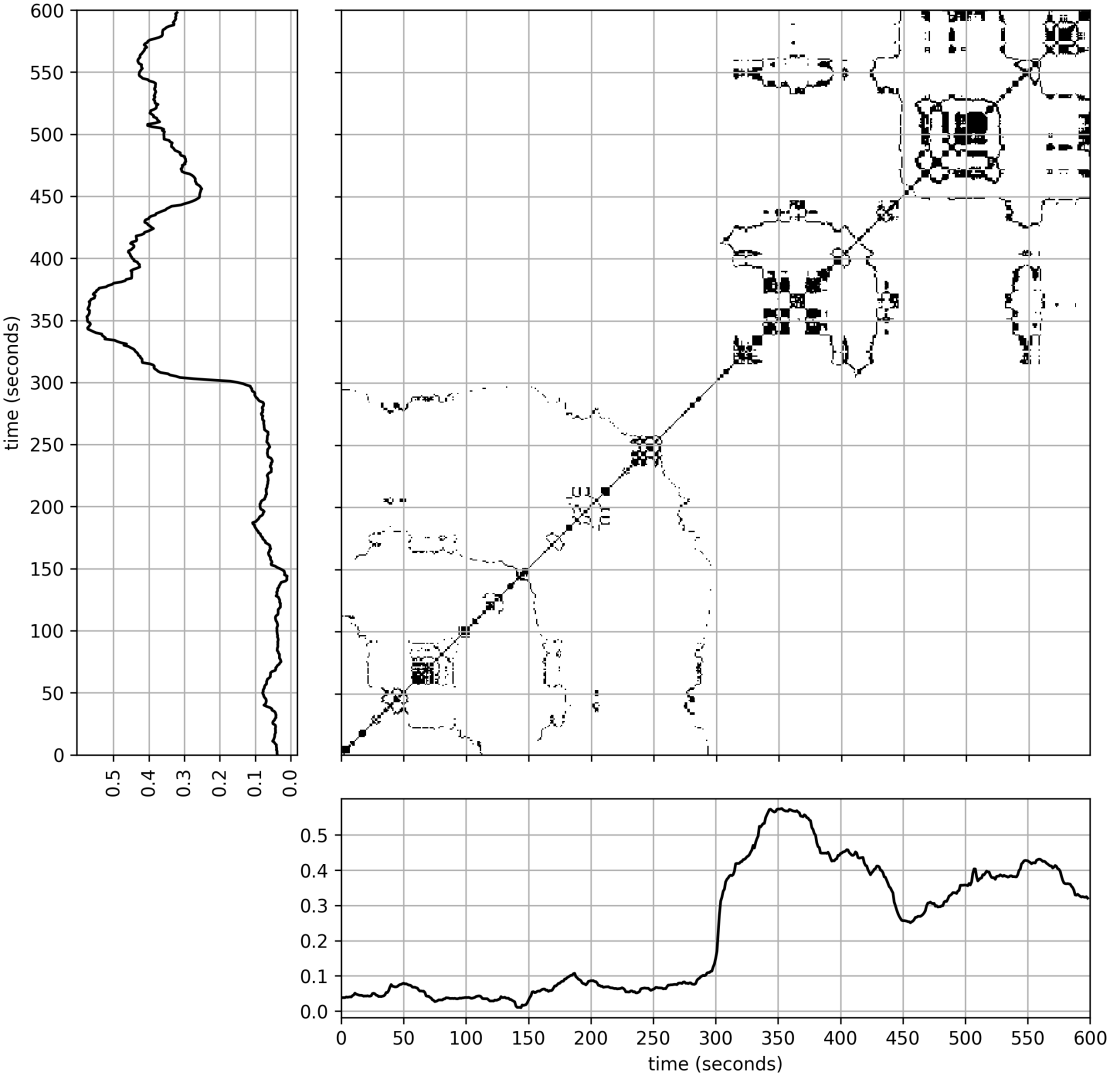
A recurrence plot based on the time-series shown on both the vertical and horizontal axis, generated with parameters: recurrence rate = 3%, *D* = 1, and τ = 1. Off-diagonal formations show periods of retained or repeated values as horizontal/vertical and diagonal shapes, respectively. This is not included in recurrence quantifier calculations.

Recurrence quantification analysis (RQA) is a non-linear method for characterizing individual time-series (Thiel et al., 2002; Zbilut et al., 2002; Webber et al., 2009), being robust against non-stationarities and noise. The method has been widely applied in many fields (see, e.g., Marwan et al., 2007), and in analyzing dynamics of physiological signals in social interaction (Fusaroli et al., 2014; Guevara et al., 2017; Xu et al., 2020). RQA is based on recurrence plots, which visualize temporal repetitions (recurrences) of values as points on a time–time coordinate system. This visualization is depicted in Figure 3 for our data. That is to say, both the horizontal and vertical axis take values of the time values of a time-series, and thus a repeated sequence appears as a diagonal line. Analysis of the distribution of these points and especially the diagonal lines they form, can yield a variety of interpretable quantities through RQA.

Often the first step of this technique is time delay embedding (Webber and Zbilut 2005), aiming to reconstruct the state space dynamics of higher dimensionality, lost by considering only a scalar time-series, through supplementary delayed coordinates (Takens, 2006; Goswami, 2019). This is achieved by successively delaying the time-series by some value τ , for a number of times D , called the delay, and embedding dimension, respectively. Embedded recurrence plots thus do not consider as a recurrence only a single value repeating, but a sequence, D of them separated from each other by τ . An embedding dimension D>1 may not itself be stationary however (Brick et al., 2017), as the sequence of recurrence might change its character in a non-stationary series – a feature that RQA without embedding ignores.

For continuous time-series, what constitutes two values being “the same” is not as straightforward as for discrete series. Here a radius E is considered, by which we consider two values to be recurrent if $\left(x\left(t_{1}\right)-x\left(t_{2}\right)\right)<E$ .

Once the parameters have been chosen, the simplest recurrence quantifier obtainable from recurrence plots is the recurrence rate ( RR ), which is the percentage of points that fulfill the condition $\left(x\left(t_{1}\right)-x\left(t_{2}\right)\right)<E$


$$RR=100\times \frac{N_{rec}}{N_{p}},$$

where $N_{rec}$ is the number of recurrent points and $N_{p}$ is the number of points on the recurrence plot in total. Based on this ratio, many quantifiers can be defined, of which we employ three:


$$DET=100\times \frac{N_{diag}}{N_{rec}}\mathrm{(Determinism)}$$

$$ENTR=-\overset{N_{p}}{\underset{l=l_{\min}}{\sum }}p\left(l\right)\;\log_{2}\left(p\left(l\right)\right)\mathrm{(Entropy)}$$

$$LAM=100\times \frac{N_{vert}}{N_{rec}}\mathrm{(Laminarity)}$$

The first, determinism ( DET ) describes the proportion of recurrent points that form diagonal lines, i.e., not just repeating values but a sequence that is repeating. $N_{diag}$ is the number of recurrent points forming diagonal lines. It’s worth pointing out that the minimal number of points required to consider a diagonal can vary, but mostly is chosen as the minimal possible, i.e., 2. The second, entropy (ENTR) describes the complexity of the deterministic structure in recurrent points. As p(l) is the probability that a diagonal line has length l , its entropy quantifies the complexity of a time series - higher values indicate a wider distribution of line lengths and translates to the deterministic segments being more varied in their duration. How deterministic a system is, is another thing, as DET only considers the number of points that form diagonal lines, irrespective of their lengths. Lastly, laminarity (LAM) is analogous to DET, but counts the percentage of recurrent points comprising vertical line structures, $N_{vert}$ being the number of points forming such lines. Thus, LAM describes a rate of stagnancy, periods of no change (within the radius threshold) in the time-series. In fact, for an auto-recurrence plot, meaning recurrence analysis on one time-series with itself, vertical lines are the same as horizontal lines, simply at different perspectives.

To calculate the quantifiers, RQA parameters must be chosen, and for this purpose, many methods have been used in the past. The embedding dimension D can potentially be estimated by the method of false nearest neighbors (Kennel et al., 1992), however this also needs some parameter fitting. The delay τ can potentially be estimated as the first minimum of either the auto-correlation or mutual information functions, but not all time-series will provide a reasonable value through such methods. There is also evidence that it is not always important to set these parameters, especially if reconstructing the nonlinear state space is not of interest (Iwanski and Bradley, 1998; March et al., 2005). This can be due to the RQA outputs remaining independent with respect to D . Furthermore, if their numerical value does not have to be the “true” value, meaning if only comparisons are of interest, RQA without embedding ( D=1 ) performs just as well on experimental series (Iwanski and Bradley, 1998). Such an approach has yielded satisfactory results in previous research (Piskun and Piskun, 2011; Arunvinthan et al., 2020; Soloviev et al., 2020) and was found suitable for our data as well.

Lastly, the threshold radius E , defining recurrences can be chosen through empirical or statistical considerations. Larger radii yield more recurrent points, possibly cluttering the recurrence plot, making interpretation difficult and the obtained results uninformative. Among multiple suggestions, a popular one is choosing E that yields a low ( 1 – 5% ) recurrence rate while the latter scales with E linearly in the log–log sense (Webber and Zbilut 2005; Wallot and Leonardi, 2018). Another suggestion for facilitating comparisons between diverse signals is instead fixing the recurrence rate, letting E vary accordingly (Curtin et al., 2017; van den Hoorn et al., 2020).

We utilized RQA as a measure of temporal dynamics inside windows of signals, to be used in a machine-learning classifier model. For the RQA parameters, we choose D=τ=1 in all recurrence quantification analyses. For E , in order to compare groups we employed an algorithm to find a suitable E conditioned on fixing the recurrence rate at 3% , as this yielded good model accuracy for our data.

The main reason to employ RQA derives from its malleability by these parameters. Indeed, the recurrence plots and values for recurrence quantifiers change when different parameters are used, but to what extent depends a lot on the system under study. The main important issue here is that the results are interpretable and comparable. For example, when working with N sets of a single measurement dimension, say a single individuals smile patterns on different occasions, then a fixed recurrence radius makes sense, as we would assume the persons underlying characteristics to be consistent, i.e., the radius of recurrence reflects their personality or related properties. This is not comparable however to other people, whose baseline for repetition of a smile can be different and while within their own dataset, a single radius is suitable, the radius is not the same as for the first individual. That is why a fixed recurrence rate is fixed instead, enabling everyone to fit with their own radius, while retaining a comparable set of quantifiers based on the percentage of repetitions. As for the value of 3%, the general best practice is to set this value as low as possible while retaining a vivid recurrence plot. For a stricter rule of thumb, the scaling between quantifier values and the recurrence rate should be linear. Thus, increasing the rate to 4% would increase or decrease the entropy, determinism, etc. linearly. In our dataset 3% was well in a linear regime of scaling and also a small value, enabling recurrence plots to be descriptive and thus the quantifiers as well. There are unfortunately no more rigorous methods to set the value of the recurrence rate, which is common for model-free tools of analysis, and a certain level of expertise must be employed.

In terms of the dimension and delay of embedding being set to 1, meaning no embedding is used, this is due to us only being interested in comparisons of recurrence patterns, not a “true” value of any of the quantifiers. Embedding is used to reconstruct the system’s true phase space and is necessary if the study of the latter is of interest, however, the lack of embedding does not take away from a uniform comparison in a lower, non-reconstructed phase space. For further clarification, we refer the reader to the references cited above, at the respective parameter descriptions.

We carried out RQA in a non-overlapping windowed framework for the whole audio drama, with a window length of 20s, yielding 600 data points. With RQA we address the second hypothesis about the data and phenomenon at hand – that the SP group shows richer, more unique dynamics than the SS group. With RQA we could capture the dynamic and non-linear patterns of behavior for individual signals via determinism, entropy and laminarity measures. Then, utilizing these quantifiers as features for a classification algorithm, the ML model can better identify in what manner and how much the quantifiers differ between the groups helps us answer the proposed question in quite large detail. This has the double purpose of also providing an estimate of predictive power for RQA of facial features alone, in the context of dyadic interaction. We tested this hypothesis by training a binary classifier to separate SS and SP signals.

#### 2.6.3. Gradient boosting classification

We employed ML to evaluate information content of physiological signals related to the presence of another person, utilizing the CatBoost gradient boosting classifier (Prokhorenkova et al., 2018). The aim of this analysis was to see if dyadic interaction leaves an imprint on an individual’s response signals, identifiable without the need to compare to the responses of others, as is the case for ISC. While there can be many predictors derived from these signals, we employed RQA, which yields single-valued quantifiers for features of determinism, entropy and laminarity, that are capable of capturing nonlinear properties of the underlying time-series. Furthermore, RQA quantifiers are interpretable and simple in their design, making them useful in not only classification but giving them a descriptive dimension.

CatBoost relies on building an ensemble of models in sequence using the whole data, where each model reduces the previous model’s error. While there are many different gradient boosting algorithms applicable for similar goals, CatBoost features symmetric decision tree generation, resulting in faster computations and less risk of overfitting (Dorogush et al., 2018), and sees lots of application in scientific inquiry (Hancock and Khoshgoftaar, 2020). The algorithm also readily provides an importance score to all prediction features, which indicates how much on average the prediction accuracy changes, if the feature value changes. We normalized this metric between 0 and 100. It gives an intuitive overview of the impact any given predictor has on the model’s performance, and we used it to interpret the latter to a degree, which we use in the context of recurrence quantifiers. We employed the open-source Python implementation via the package ‘catboost’ available at https://github.com/catboost. The label for the classifier was group belonging (i.e., SP and SS groups) and the predictor variables were RQA quantifiers (determinism, entropy, and laminarity) of data segments. With this analysis, we could test our third hypothesis related to the identification of the group identity of a subject on the basis of signal properties.

## 3. Results

In the following sections, we report our results regarding three data sources: Gaze direction, and two facial action units (AU2, AU12) which relate to facial signals. These three signals can be linked to socially motivated facial movements, here, listening to an audio drama with a pair against listening to it alone. Next, we discuss our reasoning for selecting these three signals. Our analysis codes can be found in the following repository: https://github.com/SanderPaekivi/ISC_RQA_Toolkit.

### 3.1. Initial analyses and selection of eyebrow, smile, and gaze responses

To identify facial behavior/expressions of individual participants, the Initial analysis was carried out on FACS signals from all subjects: AU1, AU2, AU4, AU5, AU6, AU9, AU12, AU18, AU25, Gaze Angle. However, in this paper, for the purpose of clarity regarding the applied methods, we specifically focused on reporting the results related to three facial modalities, AU2, AU12 and the Gaze Angle data. This choice for these three facial signals was motivated by considering overlap and performance between signals that are gathered from the same facial area. As an example, in FACS the AU2 signal informs about the movement of the Outer Brow, the *Frontalis, pars lateralis* muscle, that may be interpreted as an indication of a positively valenced, yet to a great extent automated, uncontrollable facial muscle movement. AU1 overlaps to an extent with modalities of AU1 and AU4 (Inner Brow Raiser and Brow Lowerer respectively). Considering their performance with the above-described analysis methods then, the descriptor that most clearly differentiated between SS and SP groups was chosen.

**Figure 4:**
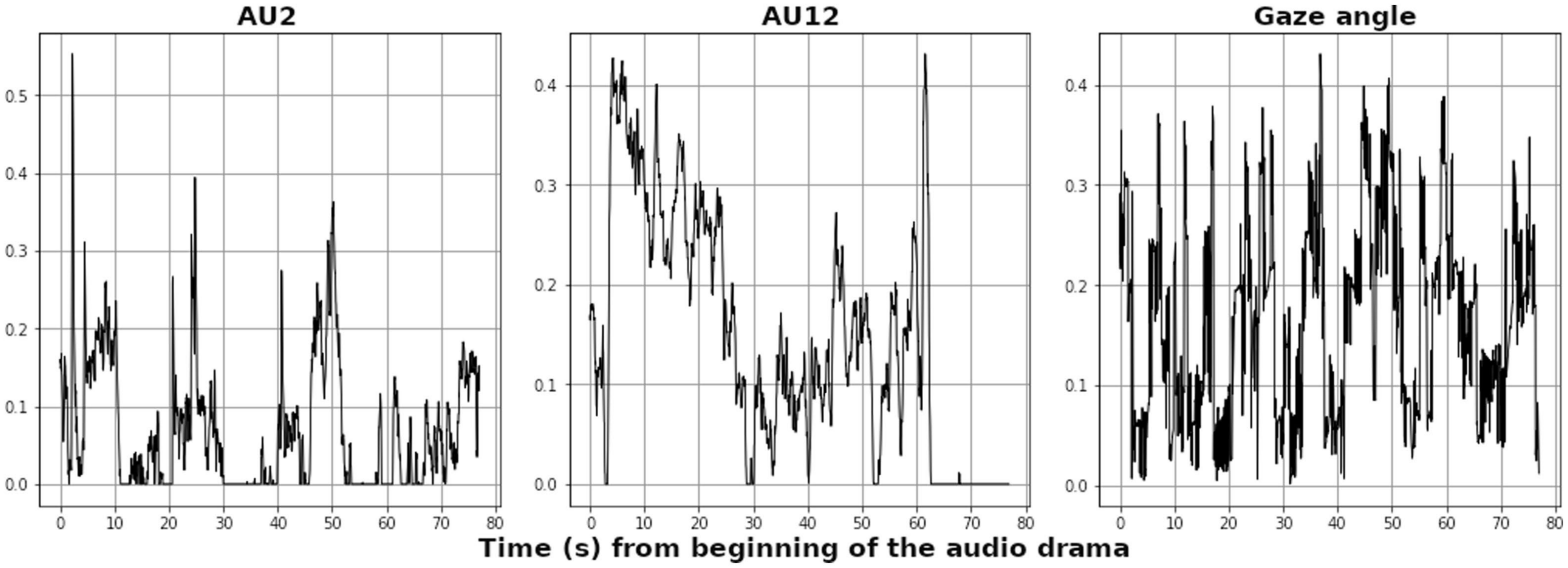
Examples of the main analyzed modalities, AU2 (“eyebrow”), AU12 (“smile”) and gaze angle from a representative individual (sp07-1), taken for the first 80 s from the onset of the audio drama. All signals are in arbitrary units given by the face tracking apparatus, and scaled between 0 and 1, reflecting the magnitude of the participants’ motion.

Regarding AU5 through AU9, they were not found informative with ISC and RQA in differentiating between paired and single subjects. Similarly, as AU12, AU18 and AU25 all relate to the motion of the mouth, AU12 was found to best track the dynamical facial expressions that can be related to the initiation of positive social contact. From henceforth we refer to the chosen signals, AU2, AU12 and Gaze Angle, as the “eyebrow-,” “smile-” and “gaze” responses, respectively. Examples of the signals are depicted in Figure 4, demonstrating notable burstiness and zero-segments for AUs compared to the gaze signal. Note that the signals are given as normalized values from the facial tracking apparatus, reflecting the facial features movement in one arbitrary dimension. The means and standard deviations of these three signals are listed in Supplementary Tables S1, S2 for the audio clips and long narrative segments, respectively.

### 3.2. Representational similarity analysis for pairwise coupling

**Figure 5:**
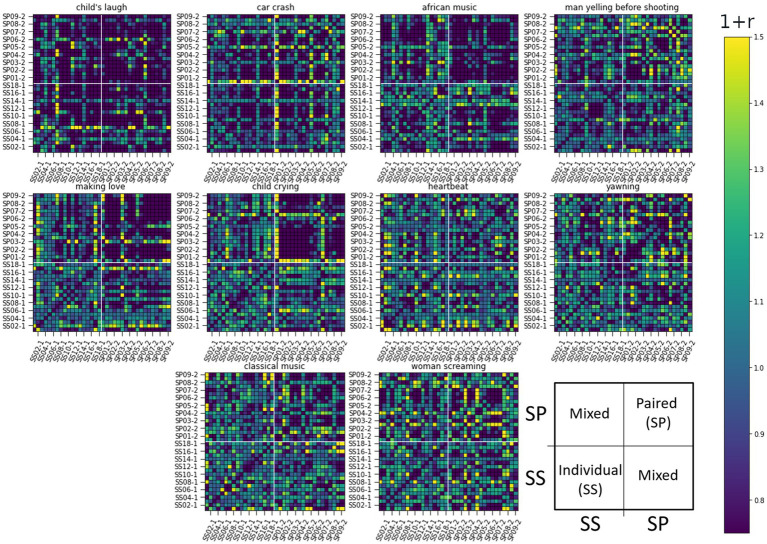
RDM matrices of the smile response (the Zygomaticus major muscle, AU12) for all 10 sound-clip stimuli. The figure presents pair-wise ISC values between all subjects. Row and column indexes are presented over one for clarity: Individual listeners ss01-1 to ss17-1 and paired listeners sp01-1 to sp17-1. Darker color corresponds to lower similarity. The arrangement of subjects and related ISC values are illustrated in the last subplot.

**Figure 6:**
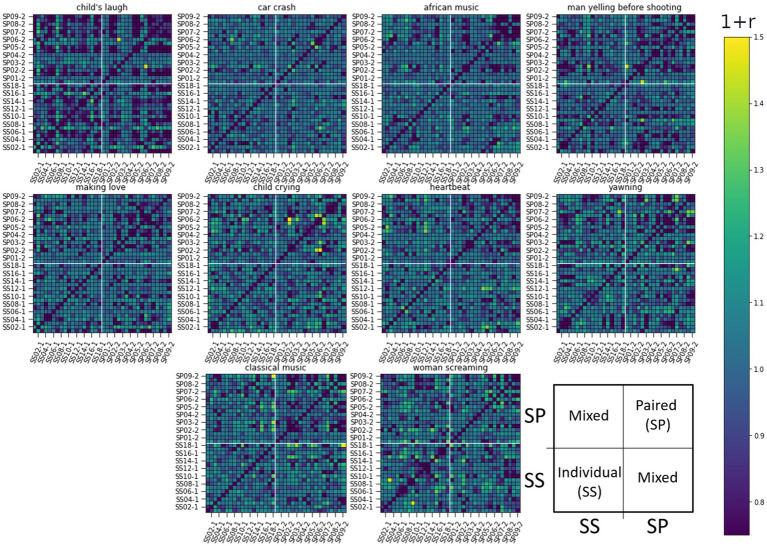
RDM matrices of the eyebrow response (the Frontalis, pars lateralis muscle, AU2) for all 10 sound-clip stimuli. The figure presents pair-wise ISC values between all subjects. Row and column indexes are presented over one for clarity: Individual listeners ss01-1 to ss17-1 and paired listeners sp01-1 to sp17-1. Darker color corresponds to lower similarity. The arrangement of subjects and related ISC values are illustrated in the last subplot.

**Figure 7:**
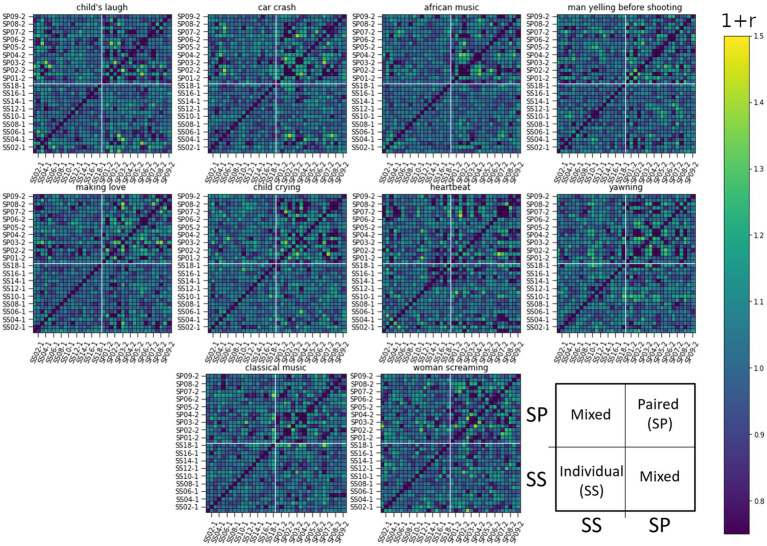
RDM matrices of the gaze response (Gaze Angle in FACS) for all 10 sound-clip stimuli. The figure presents pair-wise ISC values between all subjects. Row and column indexes are presented over one for clarity: Individual listeners ss01-1 to ss17-1 and paired listeners sp01-1 to sp17-1. Darker color corresponds to lower similarity. The arrangement of subjects and related ISC values are illustrated in the last subplot.

We calculated the ISC matrices for the 10 initial localizing segments each lasting 12 s. To capture lingering effects, we performed the analysis on 20s long non-overlapping windows, as this size proved informative. ISCs were calculated for all pairwise combinations and ordered in the RDM matrices seen in Figures 5–7.

Figure 5 depicts the RDM matrices for the smile response (the Zygomaticus major muscle, AU12 in FACS). Row and column indexes are presented over-one for clarity: starting at individual listener *SS*01-1 (label hidden), followed by *SS*02-1, followed by *SS*03-1 (label hidden) etc. Paired listeners (SP) follow a similar notation: *SP*01-1 (label hidden) and their partner is *SP*01-2. The structure of matrices is illustrated in the last subplot with mixed pairs located in upper-left and lower-right blocks of the matrix.

When observing the visualizations of these matrices, the stimuli [Child crying], [African music], and to some extent [Making love] all exhibit clear separation of ISCs between *SS* individuals (lower left corner) and SP individuals (upper right corner). These stimuli elicit different reactions in the participants in the dyadic condition compared to the individual listeners. Specifically, we see the upper right corner of the ISC matrix shaded darker, representing lower similarity. The distinct difference between the SS and SP groups shows a decrease in synchrony as defined by correlation. This can be interpreted so that the localizing sounds induce more unique responses in subjects who have a partner, while individual listeners yield a more uniform response. These unique responses, regardless of the true pair, can be assumed to emerge due to a dialogical yet silent facial interaction elicited by the drama stimulus. Such social interaction can be detected as non-correlating response signals. This is not the same as the second hypothesis, however, as correlating signals can be more or less complex (the latter is tested via RQA). The ISCs between paired and single listeners showed on average a medial response, indicative of a lack of correlation, as could be expected due to the different listening conditions.

The RDM matrices were calculated also for the eyebrow and gaze responses (AU2 and Gaze Angle) presented in Figures 6, 7 respectively. For these modalities, it is harder to visually identify strong differences in reaction between the studied groups to any stimuli in particular.

In these latter two cases, the difference of ISCs between SS and SP individuals seems to be in their homogeneity. Namely, the values of ISCs between SP individuals range from the lower to the upper bound (see top right quarter of panels), while ISCs between SS individuals show less deviation from a general mean as do ISCs between individual and paired subjects. Next, we use RSA to quantify above visual observations.

### 3.3. RSA with coupling models

To quantify the similarities of ISCs for different stimuli, we performed Kendall Tau rank correlation analysis on the RDM matrices discussed above. Furthermore, for a description of the differences between the SS and SP groups specifically, we included in this analysis two model matrices, Model-1 and Model-2 based on our first hypothesis related to synchrony differences.

#### 3.3.1. Model-1 (“the strong coupling model”)

Assume higher similarity for only true paired listeners. This means that for example, SP01-1 and SP01-2 are assumed to have an ISC value of 2 (i.e., perfect correlation), while SP01-1 and SP02-2 have a value 1 (i.e., zero correlation). This model essentially assumes that dyadic interaction presents itself in the response signals only when the true dyadic couples are compared.

#### 3.3.2. Model-2 (“the weak coupling model”)

Assume higher similarity for all paired listeners, regardless of true couples, i.e., all SP group members’ ISC value is assumed value 2. This model essentially assumes that dyadic interaction induces a shared component in response signals regardless of the specific partner (true or not).

**Figure 8:**
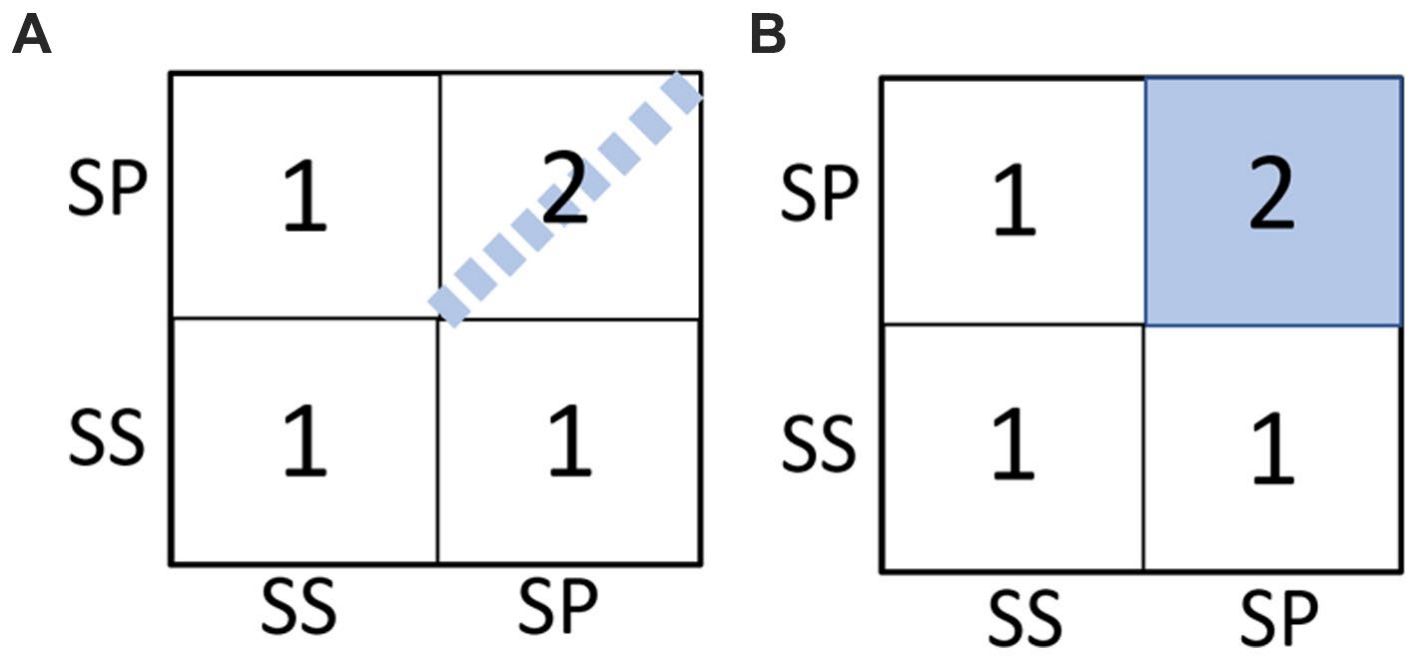
Illustration of the two coupling models. **(A)** Model-1 aka the strong coupling model (true pairs in SP) and **(B)** Model-2 aka the weak coupling model (all pairs in SP).

Above two models are illustrated in Figure 8 as RDMs.

**Figure 9:**
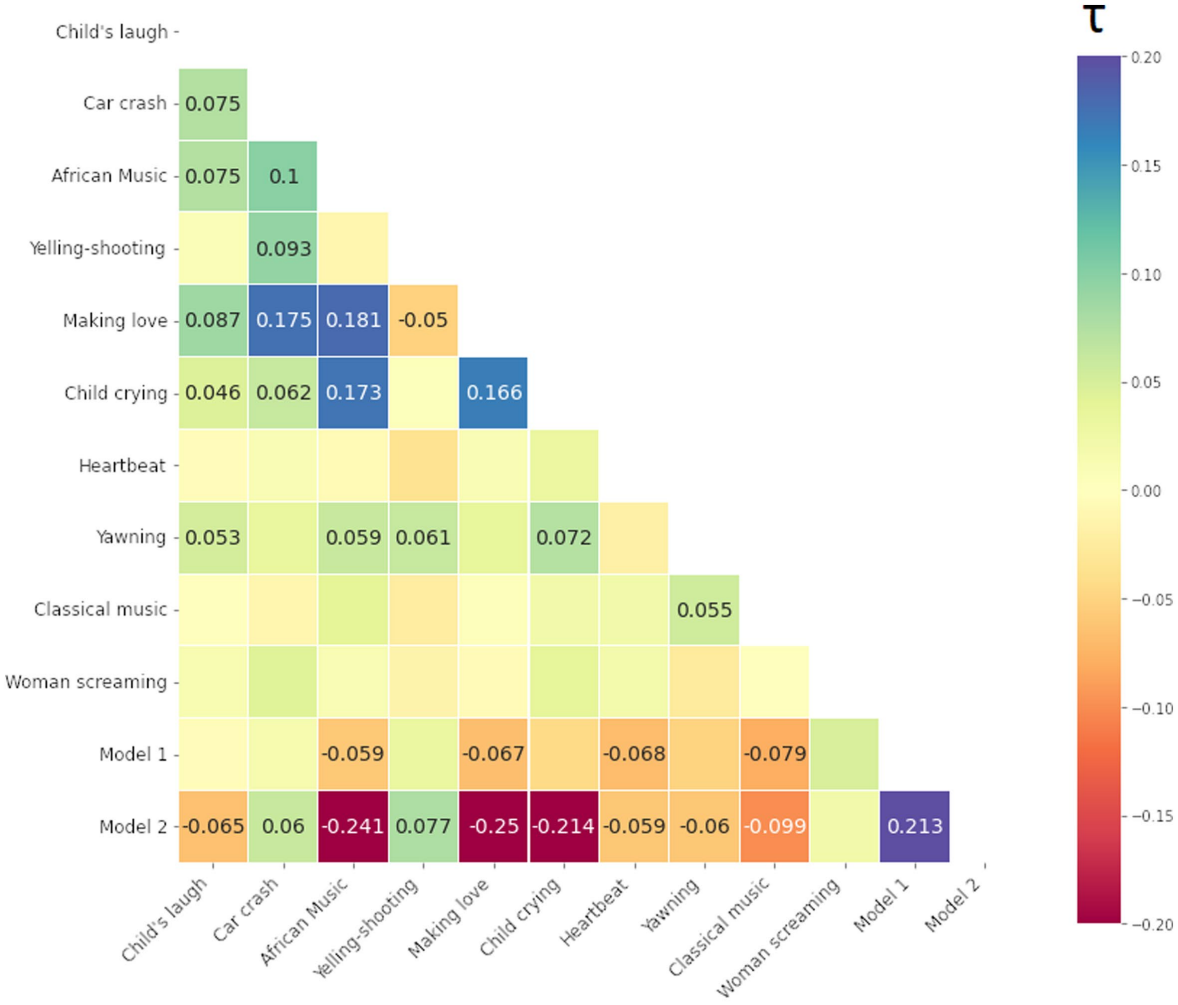
Kendall Tau correlation coefficients of RDMs for the smile response (the Zygomaticus major muscle, AU12 in FACS). Correlations between all 10 audio-clip stimuli and two hypothesis coupling models (1 being the strong coupling model). Blue color indicates more similarity, red more dissimilarity (marked with – minus). Only coefficients with statistical significance at *p* < 0.05 are shown.

**Figure 10:**
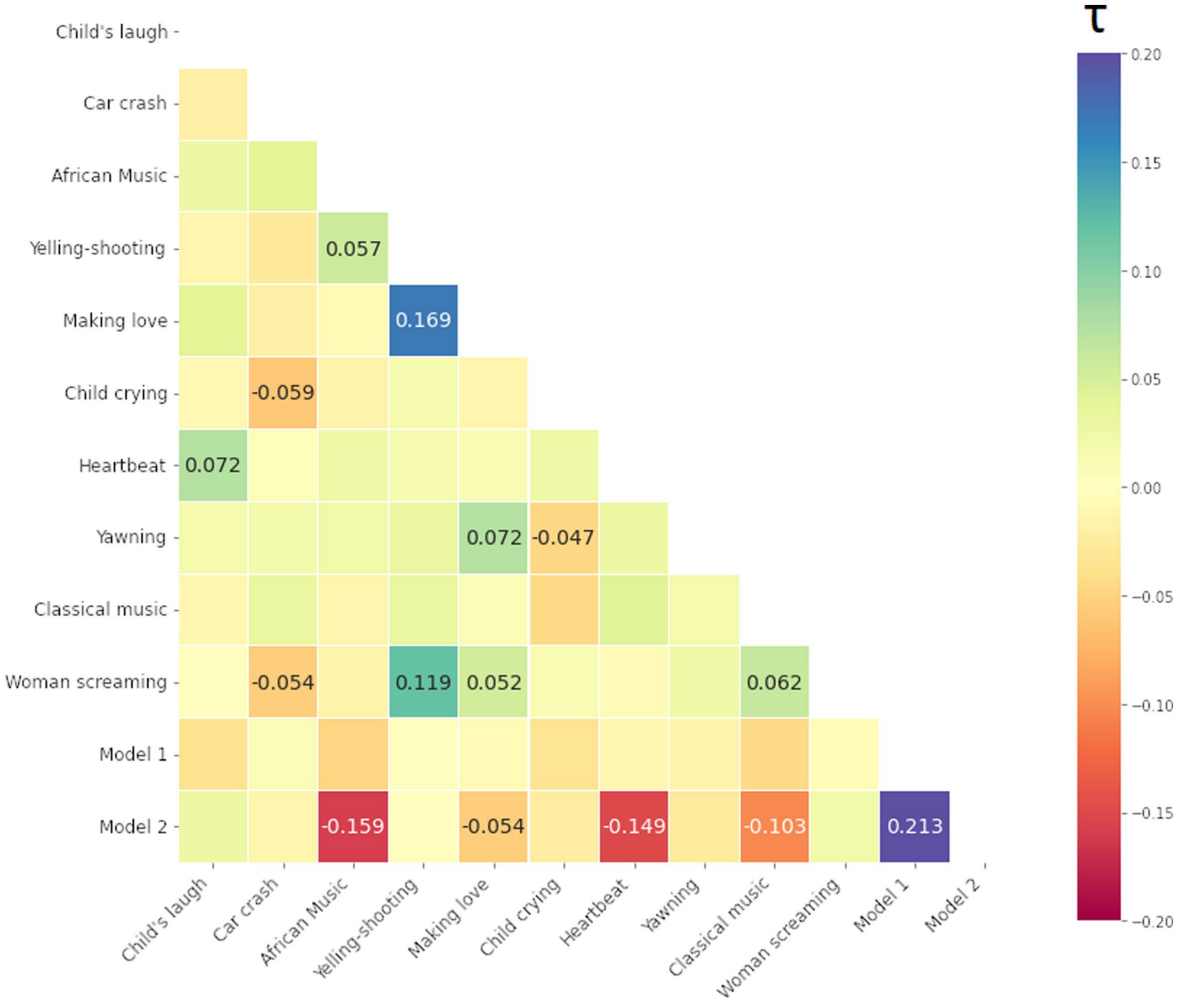
Kendall Tau correlation coefficients of RDMs for the eyebrow response (the Frontalis, pars lateralis muscle, AU2 in FACS). Correlations between all 10 audio-clip stimuli and two hypothesis models (1 being the strong coupling model). Blue color indicates more similarity, red more dissimilarity (marked with – minus). Only coefficients with statistical significance at *p* < 0.05 are shown.

**Figure 11:**
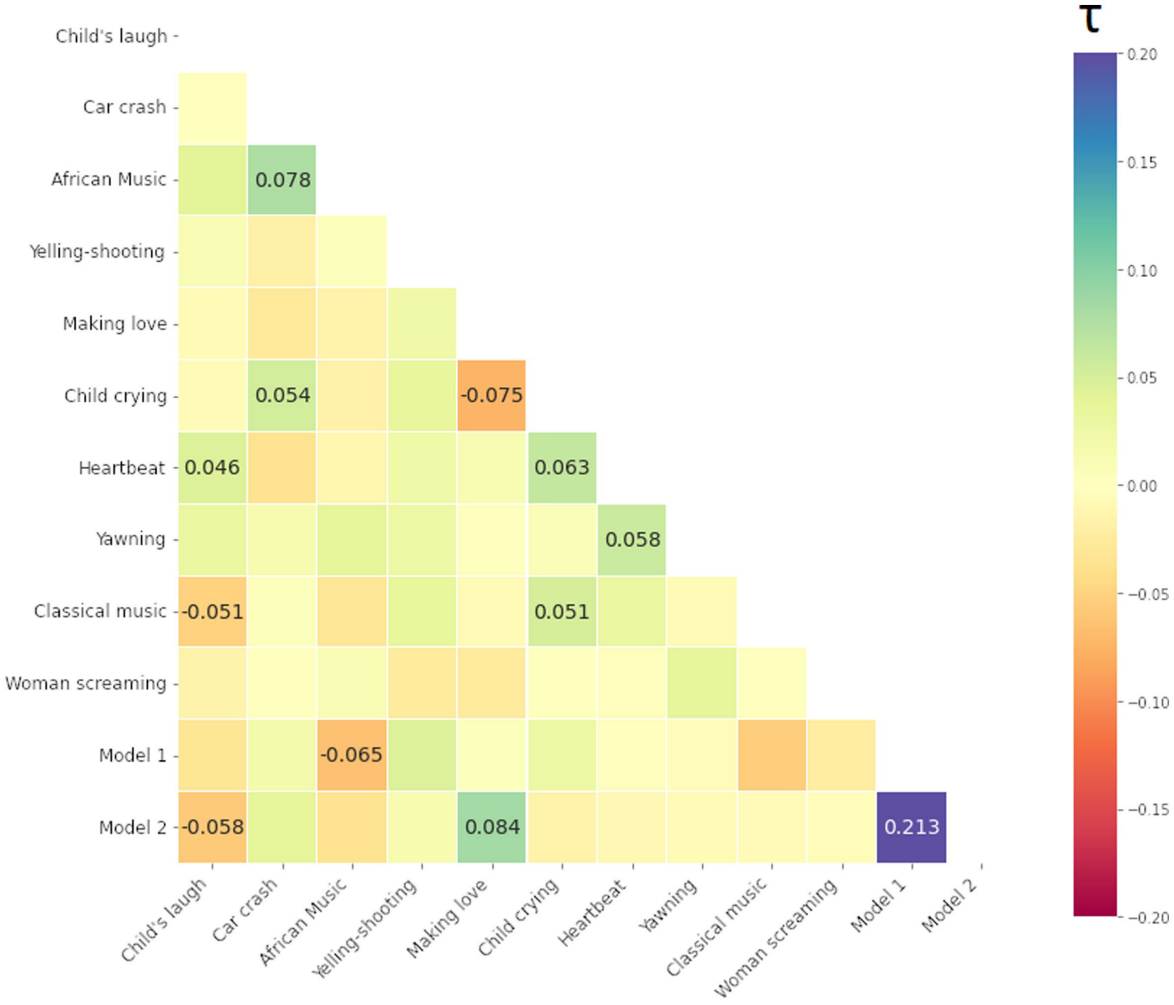
Kendall Tau correlation coefficients of RDM matrices for the gaze response (Gaze Angle). Correlations between all 10 stimuli and two hypothesis models (1/2 being the strong/weak coupling model). Blue color indicates more similarity, red more dissimilarity (marked with - minus). Only coefficients with statistical significance at *p* < 0.05 are shown.

We computed Kendall Tau rank correlations between all 10 stimuli and 2 model matrices, i.e., total (12–1) × 12/2 = 66 values (i.e., half-diagonal of symmetric matrices). The resulting Kendall Tau rank correlation matrices of the participants are presented below in Figures 9–11 for the smile-, eyebrow- and gaze responses, respectively. Correlations coefficients surpassing statistical significance *p* < 0.05 are shown.

For the smile and eyebrow responses, 6 out of 10 localizer stimuli correlated statistically significantly and relatively strongly with the Model 2, however with a negative coefficient. Firstly, this means that differences in ISC values are higher within SP subjects (true pairs or not) versus SS or mixed SS-SP pairs. That is, *regardless of specific partners in the* SP *group*, ISCs calculated between any individual from the SP group will on the whole yield different results than those calculated between individuals from the SS group. Secondly, the direction of our original assumption was reversed and in fact, individuals in the SP group respond on average *less similarly* to the stimuli than individuals from the SS group.

We can identify other connections as well. For example, in Figure 9 for the smile response we notice a relatively strong correlation between [Making love] and [Car crash] on one hand, and on the other, between [African music] and [Child crying]. These correlations represent a relationship of the pair-by-pair ISC values, and as such a positive correlation between [Making love] and [Car crash] for example tells us that their ISC matrices are significantly similar (something tentatively visible by inspection of the ISC matrices themselves). In this case, there is a significant overlap in the smile response to the mentioned stimuli, likely due to their emotionally provocative content.

The matrix in Figure 10 for the eyebrow response also shows significant correlations, however for different stimuli. We note a significant overlap in the eyebrow movement to stimuli such as [Man yelling before shooting] (shortened to [Yelling-Shooting] in the label), [Woman screaming], and [Making love].

Lastly, we present the matrix for the gaze response in Figure 11. Regarding the gaze, very few stimuli follow either Model 1 or 2, or are similar to each other. To an extent, this can arise from a stringent confidence level, but more likely is that this particular modality is not easily studied through the ISC framework. Next, we apply ISC and RSA for the signals recorded during the long narrative.

### 3.4. Sliding-window analysis of the temporally unfolding narrative

The previous analysis demonstrated the capability of ISCs to be used in analyzing dyadic interactions, so in this section we extend it to the whole audio drama, listened to by SS and SP individuals. We carried out a sliding window ISC analysis across the audio drama (27 min), seeking specific intervals where statistically significant (*p* < 0.05) Kendall Tau correlation with Model 2 (the weak coupling model) was present. The window size was selected similarly to the localizing segment lengths including their buffer times, 20 s, with a step of 1 s to capture the relevant highly influential segments. Upon generating the relevant statistics, a false discovery rate (FDR; Benjamini and Hochberg, 1995) adjustment was performed on the series of *p*-values. In order to avoid spurious results, we considered an interval to show a significant correlation with the model 2 only, if multiple windows in a row were significant.

**Figure 12:**
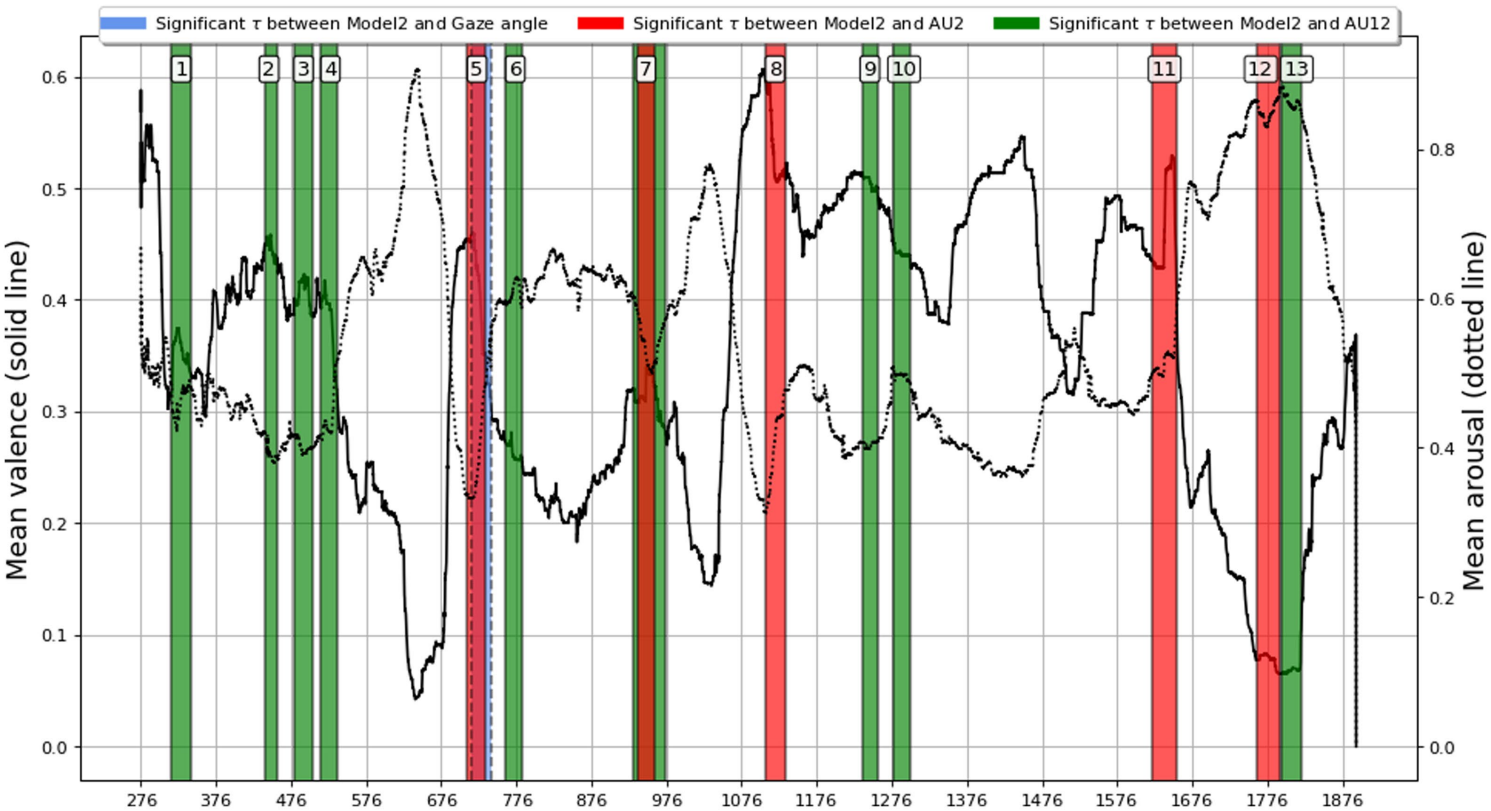
Sliding window ISC analysis across the audio drama (27 min, starting at 275 s). Lines correspond to mean valence (solid) and arousal (dotted) annotated by subjects. Colored intervals pinpoint moment with statistically significant (*p* < 0.05, FDR adjusted) Kendall Tau correlation with the weak coupling model (model 2) that corresponds to the assumption of higher similarity between paired (SP) subjects in comparison to single (SS) subjects. Model 2 assumes that dyadic interaction induces a shared component in response signals regardless of the specific partner.

All intervals with statistically significant correlations are presented in Figure 12 with color-coded and overlaid on the mean valence and arousal graphs of the audio drama, based on manual annotations of the participants. Note that because the participants annotated valence and arousal in separate listening sessions on their own, and this portion of the experiment was done voluntarily after the laboratory sessions, the N was different (*N* = 11 for valence; *N* = 14 for arousal). Here the annotated valence/arousal graph was used to describe temporally unfolding emotional content of the film, instead of applying annotations with more complicated labeling.

In Figure 12, intervals that differ notably based on modality; both seem to often precede or align with large changes in the mean valence. Also, all the significant segments of Kendall tau between the RDM and Model 2 yielded *negative* coefficients, as was visible in the RDM comparisons. Gaze Angle again showed little correspondence with Model 2, yielding only one statistically significant interval. Although this is a coarse-grained overview, averaging out a variety of subject-specific responses, the results indicate usefulness of using gaze, smile and eyebrow signals with RSA to pinpoint narrative moments associated with strong dyadic coupling. The analysis suggests that drama moments revealing socially embarrassing information or improper character behavior elicit the strongest dyadic facial interaction (see Section 4.1.2. for details).

### 3.5. Identifying dyadic interaction by machine learning on recurrence quantifiers

Lastly, we present results for the classification between SP and *SS* groups, i.e., the presence of dyadic interaction or lack thereof. The dataset was generated from the eyebrow-, smile- and gaze responses over the audio drama, split into non-overlapping 20-s segments. Such a setup yielded, for each of the 36 subjects in each modality, 3 × 3 = 9 features with total 81 segments corresponding to a total 36 × 81 = 2,916 samples for CatBoost.

The data was split into a training and a testing set in two ways. First, by randomly splitting the data with an 80% (training) to 20% (testing) ratio yielding 2,332 and 584 samples, respectively. This process was repeated 40,000 times, in order to gauge the impact of individual subject differences on the results. The optimal model parameters (L2 regularization and tree depth) were identified during training with the built-in randomized search function, and estimated in the same framework, with the optimal parameters being chosen as the average over all iterations. The second method of data splitting was based on subjects, having the training and testing set composed of different participants. The training and testing data split of 80 and 20% was maintained here as closely as possible while keeping different subjects within the two sets.

**Figure 13:**
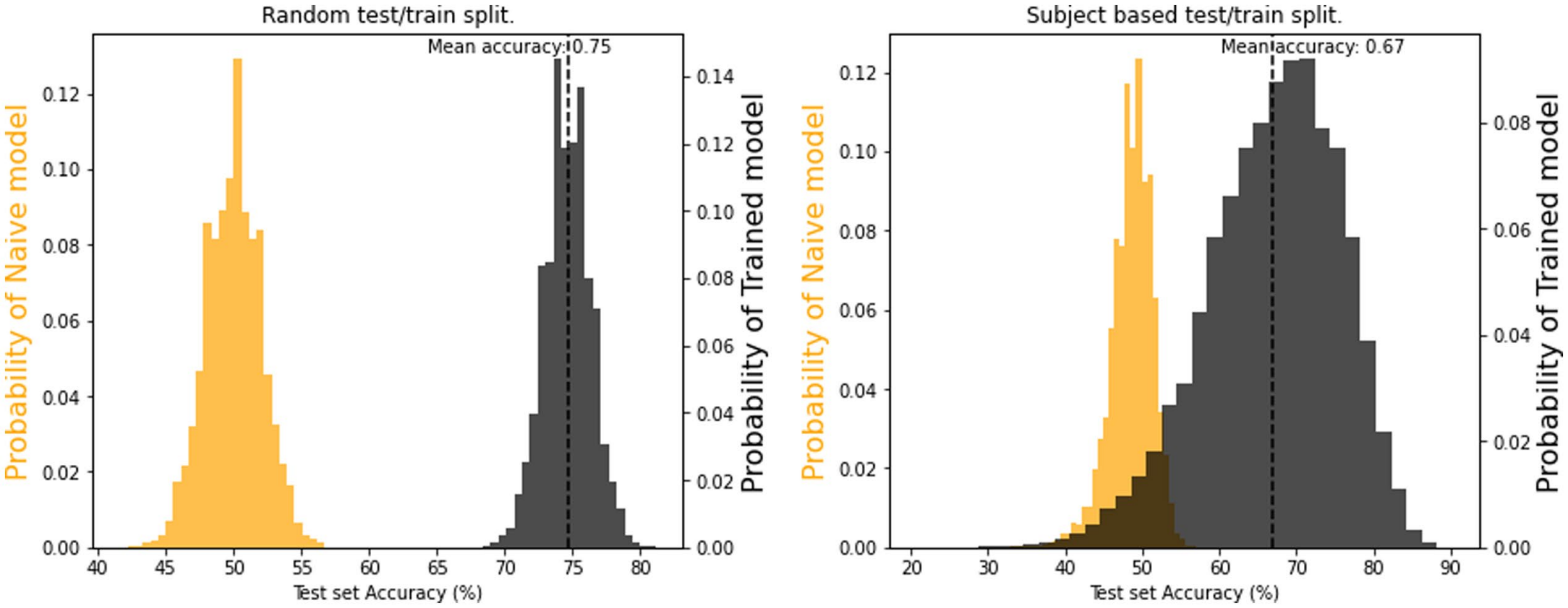
Distributions for SP/SS classification model accuracies for the test set using **(A)** random splitting and **(B)** subject-wise splitting validation strategy. The two distributions are the results for a constant-only (dummy) model (orange color) and a catboost classifier model (black color) computed with 40,000 cross-validation iterations.

In both training-test split types (i.e., random and subject-wise) the model performances were compared against a baseline (aka dummy) classifier, which was a naive predictor that always predicts the most frequent label found in the training set. The resulting distributions of accuracy percentages for both the model and a dummy classifier are depicted in Figure 13. Mean accuracies of 67% (subject-wise split) and 75% (random split) were above the chance level of 54%, indicating that models learned successfully. In other words, even short segments of our recorded physiological signals carry information on the presence of dyadic interactions, and we can classify if a specific subject belongs to the SP and SS group with better than chance probability using features obtained using RQA. This result is in line with our third hypothesis.

In Figure 13B, we notice that the subject-wise split method can generate accuracies not only overlapping with the dummy classifier, but also significantly worse than the latter. This indicates that the training and test set for those splits behave in a contrary manner to each other. The widespread, poor predictions likely relate to overfitting on the training subjects and, considering their individual differences, the test set simply does not follow the same patterns. At the same time, the subject-wise splitting procedure can also yield testing sets that perform better than any combination of the random split method, most likely containing individuals responding closer to average facial behaviors, which the model could learn from the training set individuals, irrespective of outliers.

**Figure 14:**
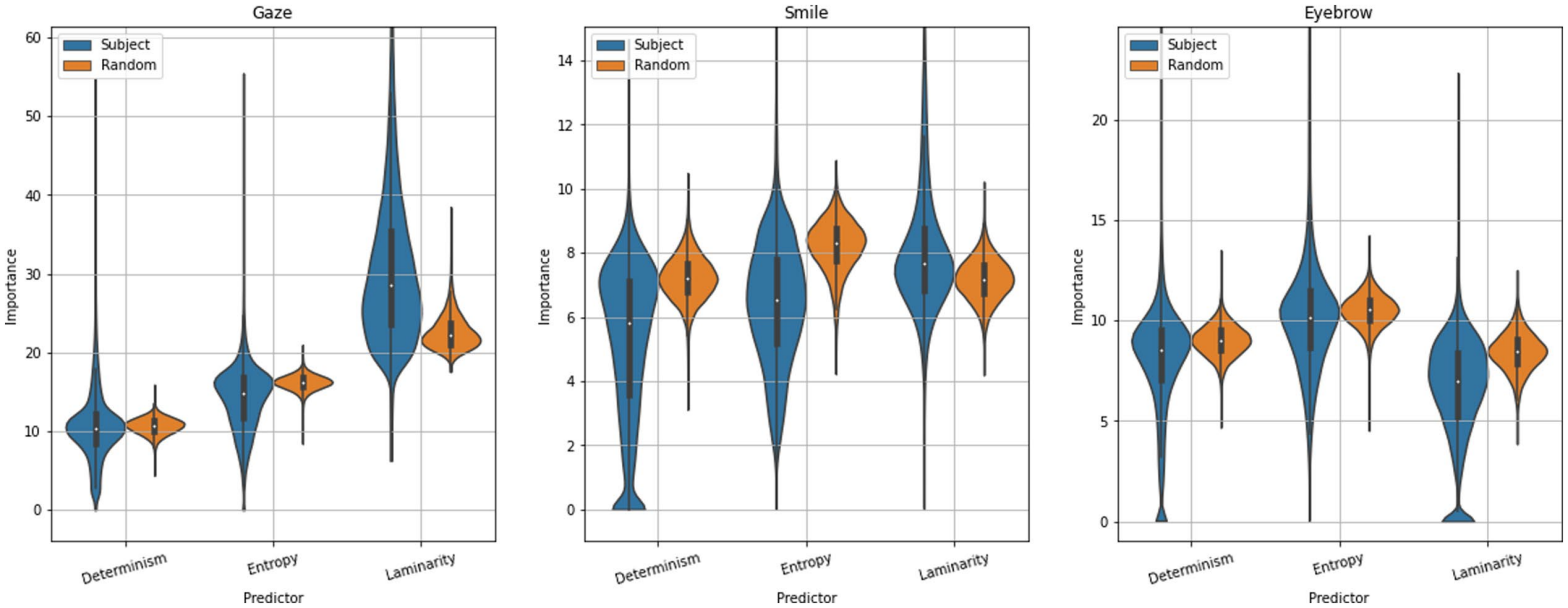
Violin plots of feature importance percentages for **(A)** gaze, **(B)** smile, and **(C)** eyebrow features in the binary classification model between paired (SP) and individual (SS) group subjects, for two train/test splitting procedures: random and subject-wise. The violin plots are bounded by the maximum 99th percentile of data within the window. Distributions were computed with 40,000 cross-validation iterations.

We further investigated the relative importance of the three features, determinism, entropy and laminarity, for the model. CatBoost feature importances indicate how much the prediction changes on average, if the feature value changes. Feature importances for the random sample-wise split and for subject-wise splits are presented in Figure 14, separately for each data type. For the three modalities, gaze was the most important with mean importance scores between 10 and 30, while smile and eyebrow are equally important at means around 6–10. Therefore, in general, all RQA features were considered relevant for the classifier (i.e., none close to zero).

When considering the random-split procedure, the following can be noticed: While gaze response carries the most importance in classifying dyadic interaction, it is based mainly on laminarity, which essentially measures stagnancy in the time-series, whereas for the eyebrow- and smile responses, entropy was the most important. As entropy is based on the recurrent points that already form diagonal lines (the basis for calculating determinism), i.e., a sequence of values being repeated, it tells us about the distribution of these sequence lengths. This in turn describes the variability in repeated sequences, as a small entropy means repeated sequences were mainly of a similar length. A high entropy smile signal for example includes many repeated smiles that vary in their motion. This can be interpreted as the complexity in the recurrent sequences being associated with dyadic interaction, while eye movement itself or lack thereof is indicative of another person being present.

There was notable variability in importance particularly for the subject-wise splitting. For the gaze response, laminarity is on average even more prioritized over other quantifiers for the subject-wise split, and laminarity also takes priority (albeit not as greatly) for the smile response. This hints at how a model that does not learn to value the entropy of the individual’s signal will make worse predictions on the whole. More information on how specifically this is the case can be found in the Supplementary Figure S2 for subject-wise splitting. Interestingly, eyebrow response retains its ordering of importance for both splitting methods, the subject-wise variant increasing the variability notably. This was also the case for further divisions by accuracy, indicating an interesting robustness of this particular signal, perhaps being more uniformly complex even for individuals whose other signals can vary widely and lead to spurious classifications. Overall, the importance scores highlight the impact of complexity in the smile and eyebrow responses for predicting dyadic interactions, in line with our second hypothesis. A detailed discussion can be found in Supplementary material.

## 4. Discussion

We report a methodic pathway for analysis of the multiplicity of psycho-physiological signals in individual and paired settings in the context of dynamically unfolding audio stimulus.

We first analyzed signals obtained for 10 sound clips from the affective sound database IADS-2 (Bradley and Lang, 2007) that is widely used in psychological studies (Yang et al., 2018), thus providing us additional support for interpretation. We found that gaze direction estimated from the video and two facial action units, AU2 (eyebrow) and AU12 (smile), provided the most robust information on pairwise dynamics. These three signals could be linked to socially motivated facial movements during listening to an audio drama. They also allowed relatively straight-forward visual validation of data analysis in terms of their social functions: Consider, for instance, movement of the gaze towards or away from the other, or simultaneous smile and/or eyebrow movements. The audio drama part allowed validating the proposed method with longer durational and dynamically more varying physiological signals. Next, we discuss our key results and their interpretations in more detail.

### 4.1. Social presence affects signal synchrony

How the social presence is shown in the analyzed data is discussed here, first, in terms of the short audio clips, and, secondly, reflected on the 27-min audio drama.

#### 4.1.1. Dyadic behaviors detected in emotional sounds

In our intersubject correlation (ISC) analysis of short localizing audio clips, the single listener groups (SS) showed uniform ISCs, representing the audio stimuli-induced component as theorized. Interestingly, we found that ISC values between any participants in the paired group (SP) were, in general, lower compared to those in the SS group, or mixed SS-SP pairs (subjects in different groups). In other words, regardless of specific partners in the SP group, ISCs calculated between any individual from the SP group yielded different results than those calculated between individuals from the SS group. This could be interpreted so that dyadic interactions do not induce a specific time-locked response but rather a communication with different dynamics of turn-taking patterns between the pairs. The latter could be assumed to consist of temporally delayed socially determined responses between the members of specific pairs, not imitation, thus yielding lower time-locked ISC. Lower ISC values for pairs that for the SS group could be due to communicative turn taking (see, e.g., turn taking in question reply setting, Bögels, 2020). In this case, one’s response to the stimuli (e.g., a spontaneous smile or a glance toward the partner) is met with a unique reaction from the other person, in turn provoking potentially further nonverbal interchange based on subtle facial expressions, i.e., dyadic reaction emotion (Sham et al., 2022).

The lower ISC values of SP than SS groups was especially evident for the modality of “smile” (AU12). This may indicate that single subjects showed a tendency to smile even alone when hearing, for example, a child laughing. This positively valenced behavior has been shown to emerge almost automatically (Stark et al., 2020), reflecting innate “caregiving instinct” in adults (Young et al., 2016). For smile (AU12), the strongest distinction between the groups when comparing the ISC results was found in the stimuli [African music], [Making love], and [Child crying]. For the eyebrow response (AU2), visually the ISC matrixes were much less distinct, however separation of SP and SS groups could be seen [Making love], [Man yelling before shooting], [Heartbeat], and to a lesser extent in others. In the case of gaze angle, a similarly weaker distinction was visible when compared to the smile response, however, [African music] and [Child crying] remain tentatively distinct, however [Making love] less so.

To handle the patterns more rigorously within the ISCs, we employed RSA with two coupling models. We also compared RDMs between stimuli. Besides finding out which stimuli produce similar results, we could also identify which matrices were entirely or almost unique in their presentation. For the smile signal, [Classical music] had a significant albeit weak similarity to [Yawning] and with the two coupling models (see Section 3.2), indicating distinguishability between two groups. The [Yawning] however was similar to other stimuli, but neither model 1 (strong coupling) or 2 (weak coupling). This could indicate that *the similarity arises from particular pairs and their interaction dynamics* rather than group differentiation. Furthermore, the stimuli [Woman screaming] seemed to be unique, following neither model nor having significant similarity to any other stimuli. Regarding the eyebrow response, a correlation with Model 1 was seen, although [African music], [Making love], [Heartbeat] and [Classical music] again fit the Model 2 assumptions, showing the separation of SS and SP in general.

The analysis of smile (AU12) provided the strongest distinction between the groups and stimuli, while eyebrow and gaze angle modality were less useful. This is possibly due to the noisy nature of the data itself and could have been expected from a visual inspection of the RDMs themselves. Furthermore, since ISC only measures linear correlation between signals, it cannot take into account any non-linear or delayed forms of synchrony that might be present. However, while not evident from our ISC analyses, all responses turned out to be informative of the presence of dyadic interaction between the pairs.

#### 4.1.2. Context-dependent dyadic behaviors detected in audio drama

The short audio clips in the IADS-2 database involve emotional situations categorized based on the emotional valence and level of arousal they elicit in participants (Yang et al., 2018). These clips, for instance, [woman screaming] or [child laughing], are effective due to their generality in terms of basic emotions, such as fear, sadness, or happiness. However, they lack time-dependent contextualizations characteristic for long durational narratives, not to mention contexts related to the lived experience of each participant. Our analysis allowed for detecting socially relevant events that reflect dyadic interactions (SP) and are not similarly present in the data from single listeners (SS).

We detected 13 moments in the audio drama (see Figure 12) where AU2, AU12, and gaze data suggested dyadic interactions between the subjects who had a pair. A description of these moments is listed in Supplementary Table S3. These moments take place in three environments, around the family dinner table (timepoints 1–4), in the Elderly Nursing Home (5–7), and at the stairway of an apartment building and an apartment in the same building (8–13). The detected dyadically active moments mainly reveal socially relevant information or behaviors that deviate from generally accepted social norms, such as infidelity, racist naming, or swearing in rage. The content of many of these 13 moments also implies internal references to previous events in the audio drama, carrying context-determining knowledge that is expected to affect reactions at later time points.

Figure 12 depicts the continuous annotations of arousal and valence by the subjects while listening again to the audio drama in their own time at home after the experiment. Interestingly, based on visual inspection, all 13 moments are located around time-locked (negative or positive) peaks of either arousal, valence, or both. Although mainly tragic and painful for the characters in the audio drama, these events could also elicit humorous, sarcastic, or even empathic responses in the subjects, this in turn, creating social pressure to share such feelings or experiences with the other person in the room. It’s plausible that besides the audio drama content itself, the enhanced memory of socially relevant dyadic interaction with the partner was occurring during those moments.

### 4.2. Social presence and complexity of recurrence patterns

The ISC allows observing correlations across multiple subjects but does not provide information on the dynamics of an individual’s data. Therefore, we employed RQA to generate descriptors of individuals’ responses that are robust against non-stationarities and also interpretable in the scope of non-linear time series analysis.

We used the quantifiers yielded by RQA as predictors in a gradient boosting binary classification algorithm (CatBoost). The aim was to see if single and paired subjects could be identified based on their individual eyebrow-, smile- and gaze properties. Using the CatBoost model, the classification was successful with mean accuracies of 67% (subject-wise split) and 75% (random split), both above chance level, thus confirming that paired setup had a fundamental effect on the autorecurrence properties of the signals. It’s noteworthy that for the subject-wise splitting procedure, where the testing and training sets were composed of distinct individuals, produced a wide range of accuracies; some lower than the chance level, some higher than the relatively tight spread of the random-split procedure. This highlights the impact of individual differences in predicting dyadic interaction from the given quantifiers and was investigated more in-depth via the relative importance of the predictors. This is discussed in more detail in the Supplementary material.

Looking at the relative importance of classifiers, the most important features were associated with gaze, and specifically its laminarity quantifier. The latter being the count of recurrent points forming vertical (or horizontal) lines describes a rate of stagnancy - periods of no change in the signal’s value (*cf.* Figure 3). Another way to describe the situation is that laminarity indicates how likely the system is to be trapped in specific states at any given time, that is to say, the response value changes slowly or does not change at all during some time interval. The mean length of these states is known as the trapping time, which describes the average time the system remains trapped in some stable state. Thus, we identified that the simple absence of movement in the gaze response is a main predictor of dyadic interaction. This result is in hindsight expectable, as another person in the room giving rise to dyadic interactions is expected to alter the movement of the participants’ eyes. However, this effect was notably stronger for the subject-wise splitting procedure. That is to say, a model trained on one set of individuals learned to over-value the simple absence of dynamic eye movement and thus underperformed in predicting dyadic interaction among individuals with a more varied range of movement in this signal. So, while the absence of eye movement is very important, it is not the be-all and end-all, as it is not solely responsible for the predictive capacity based on gaze.

For purely predictive purposes, it appears thus that gaze is behaving almost like a trigger, for whether some interaction is taking place or not. Meanwhile, more complex communication is seen in the smile and eyebrow responses, although the smile and eyebrow responses were both less important on the whole compared to gaze, the ordering of their RQA quantifiers by importance turned out informative. For the smile signal, entropy was most important for the random splitting procedure, indicating that the complexity in an individual’s smile response was what best described dyadic interaction.

Entropy in RQA describes the complexity of the deterministic structure in the data and gives a measure of how much information is needed to recover the system dynamics from the noisy and limited data (in terms of a stochastic ensemble, we have one iteration). A low entropy indicates that the length of the longest deterministic (in RQA, an “exactly” repeated) segment is short and has low variability, characteristic of chaotic behavior, while high entropy is characteristic of periodic behaviors. We stress that while this is not as impactful in terms of predictive capability, the latter can be improved by fine-tuning and the inclusion of more data and context. The primary information obtained in this manner is however that while gaze dictates well a general presence of another person in this setup, the interaction causes a noticeable uptick in periodic behaviors of different lengths in smile and eyebrow movement.

In the subject-wise splitting procedure, the classification models were not always able to learn the value of entropy in the smile signal and thus performed worse. This was demonstrated in more detail in the Supplementary material, where the importance of the subject-wise splitting procedure was further divided into high and low accuracy percentage samples (see Supplementary Figure S2). Given that the importance scores are based on the training set, the lack of weight on entropy as a predictor of dyadic interaction thus yielded lower accuracies in the prediction task.

For the eyebrow signals, no change in ordering by importance occurred when changing the train/test splitting method, with the main difference being a wider spread in the importance around a mean, and entropy being most important overall. This indicates that the eyebrow response was relatively robust in its complexity in all individuals, in contrast to the smile response. Even given the wider spread in importance, the same structure is present in the further divided plot (see Supplementary Figure S2). It shows that while individuals might significantly vary in their personality and the SP and SS groups vary fundamentally, their eyebrow movement contains a consistent amount of variability regardless.

### 4.3. Socially meaningful audio drama events enhance dyadic interaction

The experiment had two factors that were assumed to affect the physiology of the participants, namely, (1) the dramatic content of the audio stimuli which was the same for all participants independent of the listening situation, and (2) the listening situation, which was either alone or with the presence of another listener. On one hand, it has been shown that when subjects are engaged with narratives, their responses tend to align with other subjects in synchrony with the specific narrative events (Regev et al., 2013; Tikka et al., 2018). On the other hand, in the presence of other people, the physiology and behavior of the person tend to align with those of others (Holler, 2022). While the recognition of the emotional states of other persons has shown to be especially contagious, for instance, regarding anxiety (Shaw et al., 2021), or the spontaneous smile of a co-stranger (Golland et al., 2019), affective responses to stimuli may not rely on unconscious perceptual processes only but require attentive awareness of the semantic content of the stimulus (Lähteenmäki et al., 2019).

Our results showed that the behaviors of those who listened alone were more aligned with other single listeners than the behaviors of those who had a pair. The listening situation was affected by the observed facial behavior of the pair on the other side of the table. The socio-emotional events in the audio drama triggered non-verbal responsive expressions between the pairs, which altered in a dialogical manner, likely depending on the personality of each participant. One interpretation of the lower synchronization could be that the engagement with the social face-to-face interaction during audio drama may have required socially contextualized efforts that could lead to redistribution of available affective-cognitive resources, which in the case of a single listener condition would be directed solely to the engagement with the narrative. It’s also possible that dyads were doing more complicated interactions not captured by linear ISC or were simply distracted from listening to the narrative.

In face-to-face situations, following (Hasson and Frith, 2016), it may be assumed that during a physical co-presence with another person in a room, even without tasked bodily or verbal interaction, the behavior of the participants will gradually start to be dynamically coupled. In a motivated interaction or conversation situation, the co-present parties consciously take turns, adapting their behavior to the behavior of the other (Trujillo and Holler, 2023). Again, this type of synchrony could be more complicated than linear correlation used in computing ISC, hence resulting in low apparent synchrony using ISC. In our experimental situation, the two co-present participants (SPs) were instructed not to talk with each other, but only to attend to the auditory stimuli and pay attention to the other subject’s feelings on the opposite side of the table. These instructions were used as a practical matter of preventing subjects from moving, acting or speaking aloud so that it would disturb data collection, however without biasing the experimental situation. The emotional narrative was considered a more powerful driver of emotions and social dynamics than the instructions we used.

Although our findings are based on three specific facial signals, we assume that a manifestation of this socially determined feature might be observable in any other multiple signal source combinations of the dyadic group, thus allowing to separate the people with pairs (SP) from the individual participants (SS), based on their ISC values and even the individual time-series based on RQA quantifiers.

### 4.4. Limitations of the study

In this study, we concentrated on three data sources from a range of multiple data sources collected in the experiment. This was a limitation considering the possible information embedded in the other signal sources that were not used. However, we also consider this as a strength, as pinpointing and working only with the three strongest facial behavioral modalities (AU2/eyebrow, AU12/smile, and gaze) allowed us to make more direct assumptions on the functions of each modality in terms of the socially contextualized study set-up. Consequently, interpretation of the findings is straightforward, in comparison to well-known interpretation challenges related, for instance, heart rate variances, or even more so with EEG and related neural signals. Yet, the interpretation of data of easy access modalities such as a smile or lift of an eyebrow may embed *ad hoc* interpretation biases inherited from ‘folk psychology,’ introducing limitations in its one right. While our experiment revealed notable differences in signals between individual and paired subjects, additional control variables and/or stimuli are needed to further explore the origin and factors driving those differences. For example, using more or less engaging narratives and introducing other types of interactions (see Future directions) could change the relative importance of the narrative versus social interaction.

We did not quantitatively measure the level of attention of participants towards the audio narrative, instead, we relied on the qualitative questionnaire (“What kind feelings did the story evoke?”) and post-experiment discussions about the content of the story, which allowed us to ensure that all subjects recalled at least some events in the story. In our study, all SP subjects were assigned randomly with their partners instead of measuring and matching partner sympathy ratings explicitly. This may have influenced the higher variability in the partnered participants.

Finally, the number of participants was relatively small and limited to single listeners and to the pairs that were unfamiliar to one another. While the unfamiliarity in the pair condition was deliberately chosen as one of the participant selection factors, this due to the expectation that strangeness of the other person would generate sufficiently socially driven distraction to the dyadic setting compared to the single-person setting, to properly study the actual effect of unfamiliarity us such we should have expanded the paired participant group with the familiar pairs. Due to our orientation towards elaborating novel methodic pathways, the study of familiarity vs. unfamiliarity was excluded from this experiment in favor of focusing on the single vs. paired groups.

We furthermore acknowledge that additional data collection is necessary to link behavioral and personal traits with properties of signals, such as synchrony and autorecurrence. Specifically for the interpretation and analysis of recurrence quantifiers, a larger sample size can help add confidence and clarify the role of entropy vs. laminarity among the chosen modalities, based on individual differences, as current results could too strongly be influenced by outliers.

### 4.5. Future directions

We aim to eventually apply the analysis framework developed and tested here for large-scale multisource data acquired from participants’ engaging with different social activities, not only listening to a narrative. Studying recurrence properties of physiological signals during interaction is important to better understand and model human responses to long-duration narratives in social contexts. Such information is valuable in modeling and predicting human responses to audio-visually mediated or live social situations. In addition to increasing social and psychological understanding of the dynamics of dyadic non-verbal interaction between two people, such knowledge contributes to the fields of human-computer interaction and social robotics. In this study, we limited the RQA analysis to individual signals, but extensions to multiple signals, e.g., between different modalities or individuals, including joint-RQA and cross-RQA exist (Webber and Zbilut, 2005). Here we proposed to use a classification algorithm that can yield a metric of importance for predictors, but application as numerous. For example, one might be interested in developing the RQA further on specific segments, reconstructing the proper phase space with time-delayed embedding, and perhaps even introducing cross-RQA, which is again a pairwise analysis of recurrence on two series. We focused on SP/SS group differentiation rather than individual identification; nevertheless, we highlight the ethical implications inherent in developing AI models that may be capable of individual-level physiological signal detection. Consequently, careful ethical consideration would be essential for any research moving in this direction. Finally, in our setup, we evaluated signal changes with face-to-face paired listening with another person. However, numerous other potentially interesting setups presumably induce signal changes. For example, what if another person is not face-to-face, but with a 45° angle, or there are multiple persons, an animated robot, or just a display? Each of these scenarios could leave their specific fingerprints on the physiological signals.

## 5. Conclusion

The face-to-face presence of another unfamiliar person in a shared context may be a strong driver for variations in the physiological signals. The question we tackled was to what extent this social phenomenon overrides the interpersonal synchronization induced by a dramatized context. Our experimental setting revealed differences between single listeners and dyadic pairwise patterns when listening to audio drama. We concentrated on two computational methods, ISC and RQA, that can be applied in the analysis of dyadic physiological signals without the need for manual annotations.

Our ISC analysis showed that the audio stimuli induced more unique responses in those subjects who were listening to it in the presence of another person, while single listeners tended to yield a more uniform group response as it was driven by the audio drama alone. In other words, the behaviors of paired individuals correlated less with one another, compared to the single listeners. The ISC values of pairs, whether calculated on true pairs or any two individuals who had a partner, are lower than the group with single individuals. Most likely the signals generated by pairs were not driven only by the audio drama stimulus, but also by their mutual facial interactions that were unique to particular pairs.

The strongest distinction in facial interaction between the two groups (paired vs. single listeners) was found with three sound localizers containing distinguished socially charged positive or negative valences. Out of the three facial signal sources that we studied, the ‘smile’ (Lip Corner Puller, AU12) may best indicate the momentary non-verbal facial dialogue in search of agreement on responses to the shared affective context. In the 27-min long audio drama we pinpointed 13 moments with distinctive dyadic interactions between the subjects who had a pair. Using RQA, we found that recurrence patterns in an individual’s responses carry information about dyadic interactions, via a classification task, and accounting for the entropy or complexity of recurrent patterns was important for the latter, especially so for the smile response. While the gaze was mainly a laminarity-based predictor of the presence of another person, the undervaluing of entropy related to worse classifications in all cases, demonstrates the way RQA can describe nonlinear effects of dyadic interaction on one’s response signals.

Regarding the two main tools used in the analysis, RSA and RQA, we demonstrated how the more conventional RSA is indeed effective in analyzing dyadic interactions, highlighting moments of a clear division between the two studied groups. However, this method is limited by linearity and requires pairwise comparisons. RQA however applies to a single time series with non-linear properties. The two methods target different aspects of the studied phenomenon. In short, RSA tells us where differences occur as a whole, and the latter can be compared easily to hypothetical similarity matrix structures. In our case this showed a reduction of correlation for dyadic interaction, most likely indicating a nonverbal dialogical communication. RQA tells us how individual time-series differ by their non-linear recurrent properties, which were found in the study to follow the second initial hypothesis, that dyadic interaction influences the complexity of recurrent dynamics. Specifically, the novel combination of RQA and classification tasks has many further avenues to explore regarding the data. The latter, however, should be investigated in the future in the context of dyadic interaction. This work serves as a methodological first step in this direction. Our work also contributes towards modeling and predicting human social behaviors to specific types of audio-visually mediated, virtual, and live social situations.

## Data availability statement

The original contributions presented in the study are included in the article/Supplementary material, further inquiries can be directed to the corresponding author.

## Ethics statement

The studies involving humans were approved by Local institute ethics committee (Aalto University). The studies were conducted in accordance with the local legislation and institutional requirements. The participants provided their written informed consent to participate in this study.

## Author contributions

JaK, PT, and JnK participated in planning the study, collecting the data and supervising. SP, JnK, and JaK participated in analyzing the data. All authors participated in discussing results, preparing manuscript and approval of the manuscript.

## Footnotes

^1^https://archive.mpi.nl/tla/elan
