# Supplementary material for: Unraveling dyadic psycho-physiology of social presence between strangers during an audio drama – a signal-analysis approach
Source: Front Psychol. 2023 Oct 19;14:1153968. doi: 10.3389/fpsyg.2023.1153968 (PMC10622809; doi:10.3389/fpsyg.2023.1153968)
Supplement: Supplementary file 1 [file Data_Sheet_1.pdf]

Supplementary Material

(A)

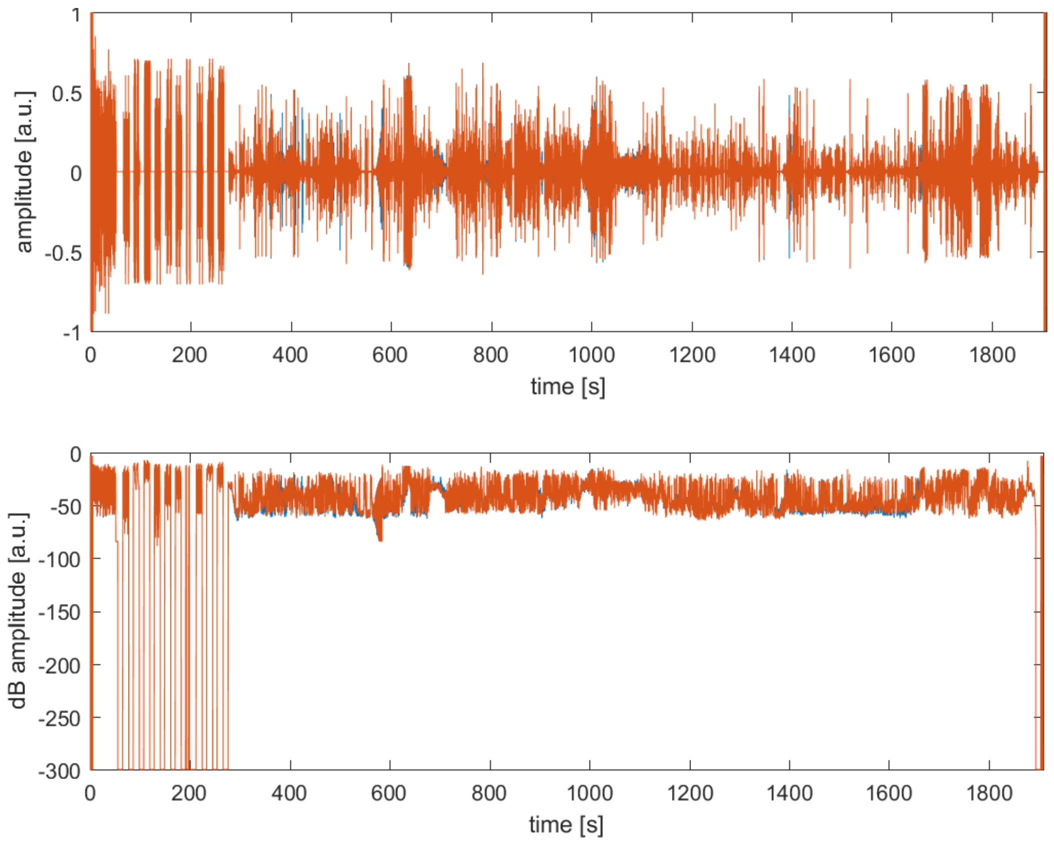

(B)

Figure S1. The whole experiment soundtrack as a waveform in (A) linear and (B) decibel scale, showing instructions phase (0-60s), localizer with 10 affective sounds (~60–300s) and the soundtrack (300s to the end).

Table S1. Statistical properties of three signals for audio clips. Each value represent mean with std in parenthesis.

Signal	Statistic	child's laugh	car crash	african music	man yelling before shooting	making love	child crying	heartbeat	yawning	classical music	woman screaming
AU12	SS Mean	0.28 (0.24)	0.13 (0.12)	0.15 (0.15)	0.11 (0.15)	0.17 (0.18)	0.12 (0.13)	0.1 (0.12)	0.1 (0.13)	0.09 (0.13)	0.08 (0.13)
	SS Std	0.12 (0.08)	0.05 (0.03)	0.06 (0.04)	0.04 (0.03)	0.08 (0.08)	0.05 (0.04)	0.04 (0.04)	0.05 (0.04)	0.04 (0.03)	0.03 (0.02)
	SP Mean	0.4 (0.14)	0.21 (0.12)	0.29 (0.14)	0.22 (0.13)	0.37 (0.16)	0.21 (0.11)	0.15 (0.12)	0.27 (0.18)	0.24 (0.14)	0.17 (0.12)
	SP Std	0.13 (0.05)	0.06 (0.03)	0.1 (0.07)	0.08 (0.07)	0.13 (0.06)	0.08 (0.03)	0.04 (0.02)	0.11 (0.08)	0.06 (0.05)	0.04 (0.02)
AU2	SS Mean	0.07 (0.11)	0.07 (0.12)	0.06 (0.11)	0.06 (0.09)	0.05 (0.08)	0.05 (0.06)	0.05 (0.06)	0.07 (0.06)	0.07 (0.08)	0.04 (0.05)
	SS Std	0.06 (0.05)	0.06 (0.06)	0.05 (0.05)	0.06 (0.05)	0.05 (0.03)	0.07 (0.04)	0.07 (0.05)	0.09 (0.07)	0.07 (0.04)	0.05 (0.03)
	SP Mean	0.03 (0.05)	0.05 (0.07)	0.05 (0.06)	0.06 (0.09)	0.04 (0.04)	0.06 (0.06)	0.06 (0.07)	0.05 (0.07)	0.05 (0.05)	0.05 (0.07)
	SP Std	0.05 (0.04)	0.05 (0.04)	0.06 (0.05)	0.06 (0.05)	0.06 (0.05)	0.07 (0.05)	0.07 (0.05)	0.08 (0.07)	0.08 (0.06)	0.06 (0.05)
Gaze	SS Mean	0.08 (0.04)	0.07 (0.04)	0.07 (0.03)	0.07 (0.04)	0.08 (0.04)	0.07 (0.04)	0.07 (0.05)	0.07 (0.04)	0.07 (0.05)	0.07 (0.04)
	SS Std	0.06 (0.04)	0.05 (0.04)	0.05 (0.03)	0.05 (0.02)	0.05 (0.03)	0.05 (0.02)	0.05 (0.04)	0.05 (0.03)	0.05 (0.03)	0.04 (0.02)
	SP Mean	0.14 (0.05)	0.14 (0.07)	0.14 (0.05)	0.14 (0.07)	0.15 (0.07)	0.13 (0.05)	0.12 (0.06)	0.12 (0.04)	0.12 (0.06)	0.12 (0.05)
	SP Std	0.09 (0.05)	0.1 (0.06)	0.11 (0.05)	0.09 (0.04)	0.1 (0.05)	0.1 (0.05)	0.08 (0.04)	0.09 (0.04)	0.09 (0.04)	0.09 (0.05)

Table S2. Statistical properties of three signals for 13 narrative segments depicted in Figure 12. Each value represent mean with std in parenthesis.

AU12 Statistic

Annotation	SS Mean	SS Std	SP Mean	SP Std
**Negative/ Footsteps/ Cutlery**	0.05 (0.07)	0.02 (0.02)	0.11 (0.1)	0.04 (0.02)
**Positive/ Humming/ Melody**	0.07 (0.08)	0.04 (0.03)	0.09 (0.09)	0.04 (0.05)
**Happy/ Amused/ Humming Melody**	0.1 (0.09)	0.06 (0.05)	0.15 (0.13)	0.08 (0.06)
**Car Doors and Noize**	0.06 (0.07)	0.04 (0.03)	0.08 (0.08)	0.03 (0.02)
**Hospital/ Negative**	0.06 (0.09)	0.05 (0.06)	0.08 (0.1)	0.04 (0.03)
**Hospital/ Negative/ Rage/ Mocking**	0.05 (0.08)	0.04 (0.04)	0.05 (0.05)	0.05 (0.04)
**Doorbell Echos/ Friendly Asking**	0.04 (0.06)	0.04 (0.04)	0.05 (0.04)	0.04 (0.03)
**Traffic/ Noize/ Yawning**	0.04 (0.08)	0.03 (0.03)	0.07 (0.07)	0.05 (0.04)
**Socially Sig. Content/ Negative**	0.02 (0.02)	0.04 (0.03)	0.04 (0.09)	0.03 (0.04)

AU2 Statistic

Annotation	SS Mean	SS Std	SP Mean	SP Std
**Hospital/Angry**	0.07 (0.06)	0.08 (0.04)	0.08 (0.06)	0.09 (0.05)
**Hospital/ Negative/ Rage/ Mocking**	0.06 (0.04)	0.07 (0.03)	0.09 (0.1)	0.1 (0.08)
**Huging & Kissing**	0.06 (0.05)	0.07 (0.04)	0.12 (0.11)	0.1 (0.06)
**Church bell/ Eating/ Drinking/ Positive**	0.06 (0.05)	0.08 (0.03)	0.08 (0.07)	0.1 (0.06)
**Stairway/ Negative/ Yelling**	0.1 (0.08)	0.08 (0.05)	0.12 (0.14)	0.11 (0.05)

Gaze Statistic

Annotation	SS Mean	SS Std	SP Mean	SP Std
**Hospital/Angry**	0.07 (0.03)	0.05 (0.04)	0.15 (0.08)	0.11 (0.05)

Dramatic moment of the audio drama pinpointed by our sliding window analysis

Our 13 detected moments take place in three environments, around the family dinner table (timepoints 1-4), in the Elderly Nursing Home (5-7), and at the stairway of an apartment building and an apartment in the same building (8-13). Scenes (8-13) which are heard later in the audio drama rely heavily on the hints (cues) about 'infidelity' provided during the first scene at the dinner (1-4). Moments (5-7) in turn, build more on external facts outside of the audio drama context, for instance, improper racist language revealing conservative attitudes towards immigrants by a hospitalized Finnish-Russian war veteran (5-6), but also to symptoms of losing the sense of time as with the old woman with Alzheimer disease (7). Description of moments are listed in Table S3.

Table S3. The short content descriptions of audio drama events identified with strong dyadic interaction based on analysis of AU2 (red), AU12 (green), and gaze (blue). The three episodes take place in following environments: Home at the dinner time (1-4), old people's home (5-7), and at the stairway of an apartment building (8-13). The marked events co-occur with peaks in dynamical annotations of mean arousal and valence by the subjects.

**AU12**	**1**	Mother and Daughter at the dinner table, Mother's voice is tired and negative when talking about Father (F). "Your father has a new project that causes so much overwork" [This can be read as a social indication for the fact that the father is having an extramarital affair.
**AU12**	**2**	F is in a good mood singing by himself -- talks only to Daughter (not to Mother), tastes the food... [It seems that he is in a "too good" mood for going to work]
**AU12**	**3**	F asks Daughter to guess the auto model of his colleague; "Mazda" the daughter replies; Father laughs, then apologizing "I guess we will be quite late..." [F leaves]
**AU12**	**4**	Daughter calls after F at the door not to forget his work bag. F returns to pick up the bag....Daughter is angry at the mother: "Why you always have to be like that...you could have at least said bye to Father"
**AU2**	**5**	Old Male Patient eats food and then swears aggressively
**GAZE**	**5**	Old Male Patient talks rudely about the food, throws it away: "tasteless… not even for pigs"
**AU12**	**6**	Old Male Patient talks rudely to Nurse with accent, referring to immigrants using racist words.
**AU12**	**7**	Old Women's voices, the other is Demented: "Men are soon coming from the fields...and nothing is ready"; In the far background Old Male Patient is shouting aggressively.
**AU2**	**7**	Old Women's voices, the other is Demented: "Men are soon coming from the fields...and nothing is ready"; In the far background Old Male Patient is shouting aggressively.
**AU2**	**8**	F 'forgot the bag" and returns to kissing L many times in the stairway of the apartment building [Love affair was implied earlier at the dinner table discussion]
**AU12**	**9**	After talking on the phone with F, his Lover (L) starts her discussion with a Boy waiting in the apartment building stairway.
**AU12**	**10**	Boy has asked if F is her boyfriend, L changes subject matter, proposed Boy should call home....

**AU2**	**11**	Boy responds to L's question about boy's father and mother: "These are childrens' things that adults cannot understand" [This humorously mirrors L's earlier saying to Boy about adult things that children cannot understand.]
**AU2**	**12**	Boy's mother is shouting to L: " …how many children do you have?" Get your own children so you don't have to infer with other people's lives"
**AU12**	**13**	Boy's raging Mother to L: "You are those who need to fuck other women's men in secret, not having your own man" Boy: "Mother take it easy, all is fine"

Individual variability of RQA in classification

By analyzing the classification models, we found there was a large amount of variance in the subject-wise split feature importances depicted in Figure 14. First, much more weight is placed on the laminarity of the gaze response - as this describes the proportion of time eyesight is at rest, its increase in importance essentially reveals the model being unable to learn deeper dynamical patterns while relying on activity in general. In terms of the smile response, laminarity is again over-valued in subject-wise split while in random splits, entropy is the most important quantifier. The entropy describes the complexity and dynamical range of the time-series, and its significant deranking in importance again reveals the model's difficulty in learning complex patterns. Interestingly for the eyebrow response, the relative rankings of quantifier importances are retained, and in fact laminarity is weakened, giving ground to other modalities (as all importances in an iteration are normalized to 100).

In the case of all these changes in relative weighting, it is of utmost importance to point out that this is applicable to the median importances and perhaps the central quantiles, but a large increase in variance accompanies them. That is to say, there are many more sub-brackets of accuracy inside of the subject-wise split samples, where different ordering is found, likely arising perhaps due to overfitting on specific individuals. The idea itself however can be demonstrated by a relatively simple further segmentation of the subject-wise split samples: samples yielding accuracies above and below the distribution mean. Variable importances for random, subject-wise with higher than mean and lower than mean accuracy are presented in Figure S2.

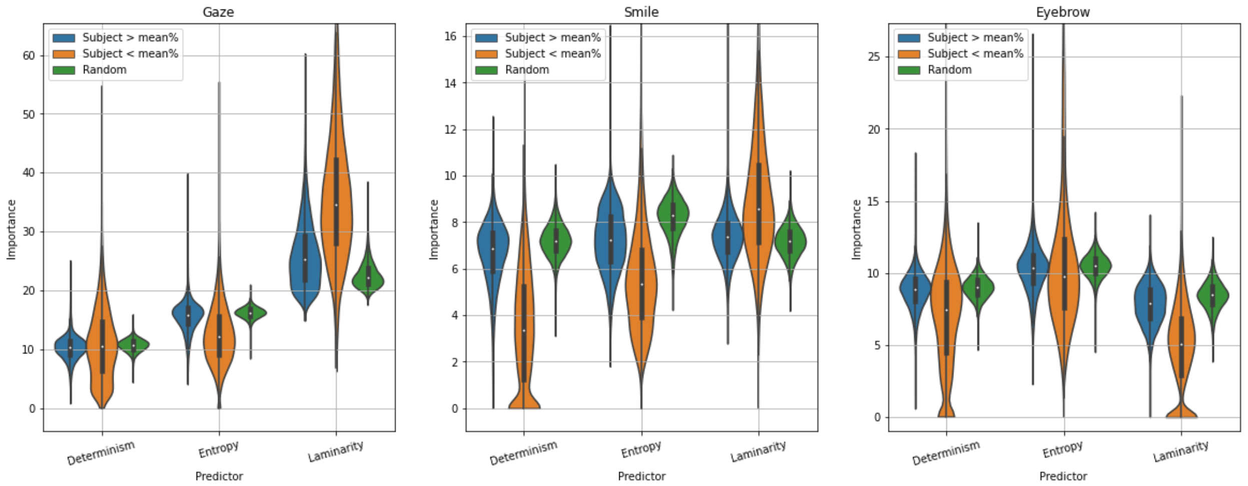

Figure S2. Violin plots of feature importance percentages for gaze, smile and eyebrow features in the binary classification model between paired (SP) and individual (SS) group subjects, for three train/test splitting procedures: random, subject-wise with samples yielding lower than mean accuracy, subject-wise with samples yielding higher than mean accuracies. The violin plots are bounded by the maximum 99th percentile of data within the window. Distributions were computed with 40000 cross-validation iterations.

The iterations that yielded better than mean accuracies follow in their feature importance the same patterns as the random splitting procedure, while the lower accuracy iterations deepen the shift in relative priorities. This detail alludes to some subjects being harder to predict by models trained on others, but not the other way around. The latter however clarifies that for the smile response, clear undervaluing of sequence repetitions (determinism) and the complexity of these repetitions (entropy) is contrasted with the overvaluing of stagnancy (laminarity). Similarly, the shift of patterns is more clear for the eyebrow response, where interestingly the complexity of repeating eyebrow movement is most important. While these are relative shifts in the feature importance within a given modality, and overall, the magnitude of gaze responses are still by and large the biggest influencer of prediction (all importance must sum to 100%), low prediction accuracy is related to an inability to learn the value of complexity in the smile response, and overvaluing laminarity in both gaze and smile response.

Prior to participation in the experiment, we asked the subjects to fill out an online version of the Empathy Quotient questionnaire, translated to Finnish (Baron-Cohen and Wheelwright, 2004) as it was considered potentially useful in separating differently performing individuals in the classification task. Interestingly, these empathy quotients available for each participant did not meaningfully separate individuals who behave differently in the scope of the classifier model. This may be partially explained by the findings of Tönsing et al (2022) that social gaze-behavior does not necessarily always show correlation with the personal traits, in their case, social anxiety. It is also noted that our focus on the complexity of the social dynamics in the shared context may render specific individual traits undetectable, or invisible. We conclude that the influence of participant's individual personal traits (such as ability to feel empathy) on the above-mentioned differences on

accuracy of the subject-wise splitting procedure can yield interesting information in regard to classification of dyadic interactions, for instance, through specific viewing windows, such as eye-gaze, smile or eye-brow behavior.

References

Baron-Cohen, S., & Wheelwright, S. (2004). The empathy quotient: an investigation of adults with Asperger syndrome or high functioning autism, and normal sex differences. Journal of autism and developmental disorders, 34(2), 163–175. doi:10.1023/b:jadd.0000022607.19833.00

Tönsing, D., Schiller, B., Vehlen, A., Spenthof, I., Domes, G., & Heinrichs, M. (2022). No evidence that gaze anxiety predicts gaze avoidance behavior during face-to-face social interaction. *Scientific reports*, *12*(1), 21332.
